# Supplementary material for: Deconvolution of spatial sequencing provides accurate characterization of hESC-derived DA transplants in vivo
Source: Mol Ther Methods Clin Dev. 2023 May 4;29:381–94. doi: 10.1016/j.omtm.2023.04.008 (PMC10209706; doi:10.1016/j.omtm.2023.04.008)
Supplement: Document S1. Figures S1–S13 and Table S1 [file mmc1.pdf]

OMTM, Volume *29*

Supplemental information

Deconvolution of spatial sequencing provides accurate characterization of hESC-derived DA transplants *in vivo*

Jana Rájová, Marcus Davidsson, Martino Avallone, Morgan Hartnor, Patrick Aldrin-Kirk, Tiago Cardoso, Sara Nolbrant, Annelie Mollbrink, Petter Storm, Andreas Heuer, Malin Parmar, and Tomas Björklund

Table S1

Supplemental Table 1: Description of used tissue sections and their animal/tissue of origin

Sample name	Animal signifier	Organism	Breed	Trans-plant age (months)	Sex	Tissue	Cell line	Cell line type
TX[L.1]	TX[L]	Rattus norvegicus + Homo sapiens	athymic nude rat	6	Female	Brain (striatal section with a transplant)	RC17	hESC derived
TX[L.2]	TX[L]	Rattus norvegicus + Homo sapiens	athymic nude rat	6	Female	Brain (striatal section with a transplant)	RC17	hESC derived
TX[L.3]	TX[L]	Rattus norvegicus + Homo sapiens	athymic nude rat	6	Female	Brain (striatal section with a transplant)	RC17	hESC derived
TX[L.4]	TX[L]	Rattus norvegicus + Homo sapiens	athymic nude rat	6	Female	Brain (striatal section with a transplant)	RC17	hESC derived
TX[L.5]	TX[L]	Rattus norvegicus + Homo sapiens	athymic nude rat	6	Female	Brain (striatal section with a transplant)	RC17	hESC derived
TX[M.1]	TX[M]	Rattus norvegicus + Homo sapiens	athymic nude rat	13	Female	Brain (striatal section with a transplant)	RC17	hESC derived
TX[M.2]	TX[M]	Rattus norvegicus + Homo sapiens	athymic nude rat	13	Female	Brain (striatal section with a transplant)	RC17	hESC derived
TX[M.3]	TX[M]	Rattus norvegicus + Homo sapiens	athymic nude rat	13	Female	Brain (striatal section with a transplant)	RC17	hESC derived
TX[M.4]	TX[M]	Rattus norvegicus + Homo sapiens	athymic nude rat	13	Female	Brain (striatal section with a transplant)	RC17	hESC derived
TX[S]	TX[S]	Rattus norvegicus + Homo sapiens	athymic nude rat	15	Female	Brain (striatal section with a transplant)	RC17	hESC derived
SN[1.1]	SN[1]	Rattus norvegicus	athymic nude rat	NA	Female	Brain (midbrain section with substantia nigra)	NA	NA
SN[1.2]	SN[1]	Rattus norvegicus	athymic nude rat	NA	Female	Brain (midbrain section with substantia nigra)	NA	NA
SN[2.1]	SN[2]	Rattus norvegicus	athymic nude rat	NA	Female	Brain (midbrain section with substantia nigra + cortex)	NA	NA
SN[2.2]	SN[2]	Rattus norvegicus	athymic nude rat	NA	Female	Brain (midbrain section with substantia nigra + cortex)	NA	NA
SN[2.3]	SN[2]	Rattus norvegicus	athymic nude rat	NA	Female	Brain (midbrain section with substantia nigra + cortex)	NA	NA
SN[3.1]	SN[3]	Rattus norvegicus	Sprague-Dawley	NA	Female	Brain (midbrain section with substantia nigra)	NA	NA
SN[3.2]	SN[3]	Rattus norvegicus	Sprague-Dawley	NA	Female	Brain (midbrain section with substantia nigra)	NA	NA
IHC analysis	TX[4]	Rattus norvegicus + Homo sapiens	athymic nude rat	15	Female	Brain (striatal section with a transplant)	RC17	hESC derived

Figure S1

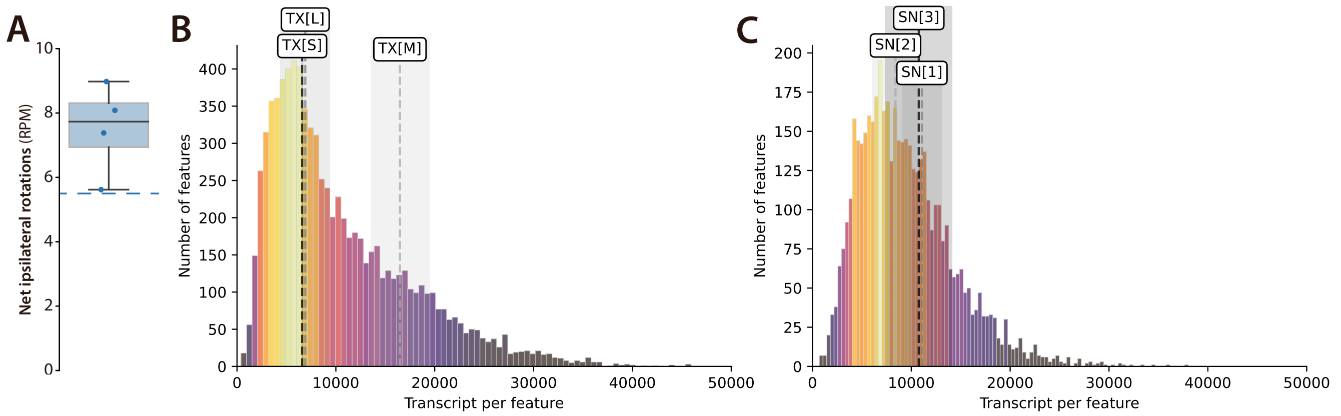

Figure S1: (a) d-Amphetamine-induced rotation of 6-OHDA lesioned animals prior to transplantation. Dashed line represents the inclusion cut-off. (b-c) Histograms of transcript count distribution per feature in (b) grafted tissue sections (TX[**S**], TX[**M**], TX[**L**]) and (c) the substantia nigra sections. Dashed lines denote sample mean per animal and the gray box one standard deviation from that mean.

Figure S2

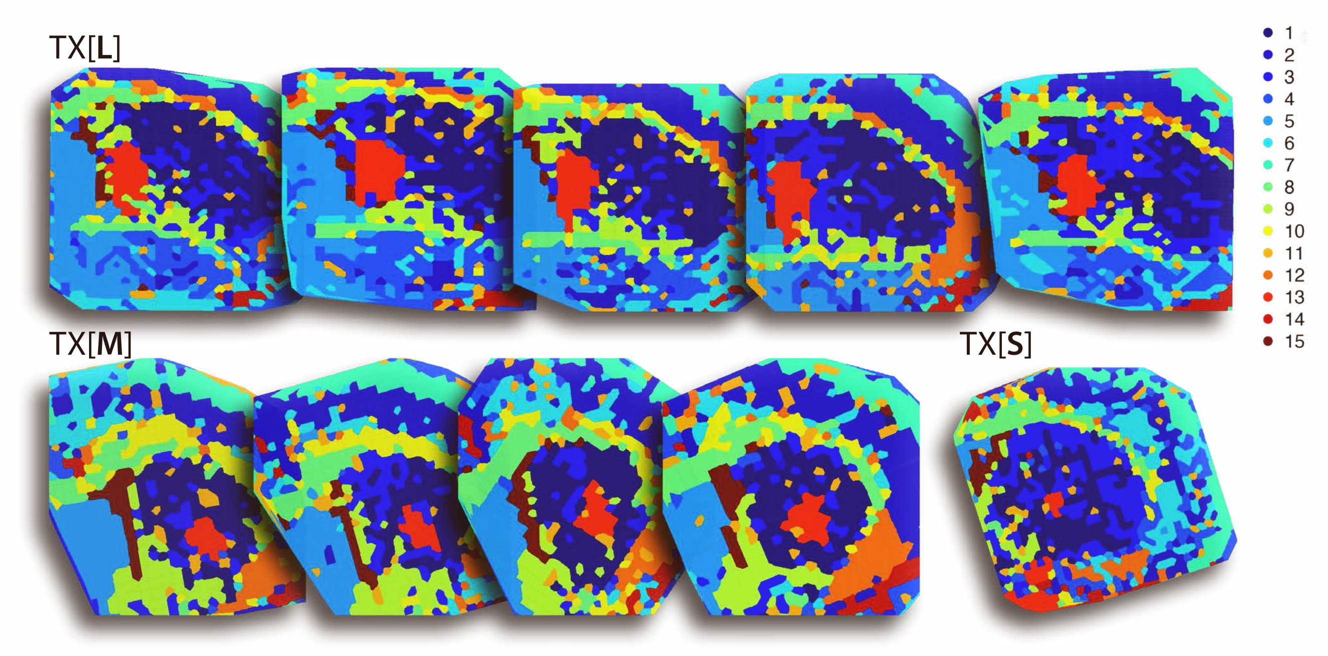

Figure S2: Seurat cluster assignment for grafted tissue sections (TX[**S**], TX[**M**], TX[**L**])

Figure S3

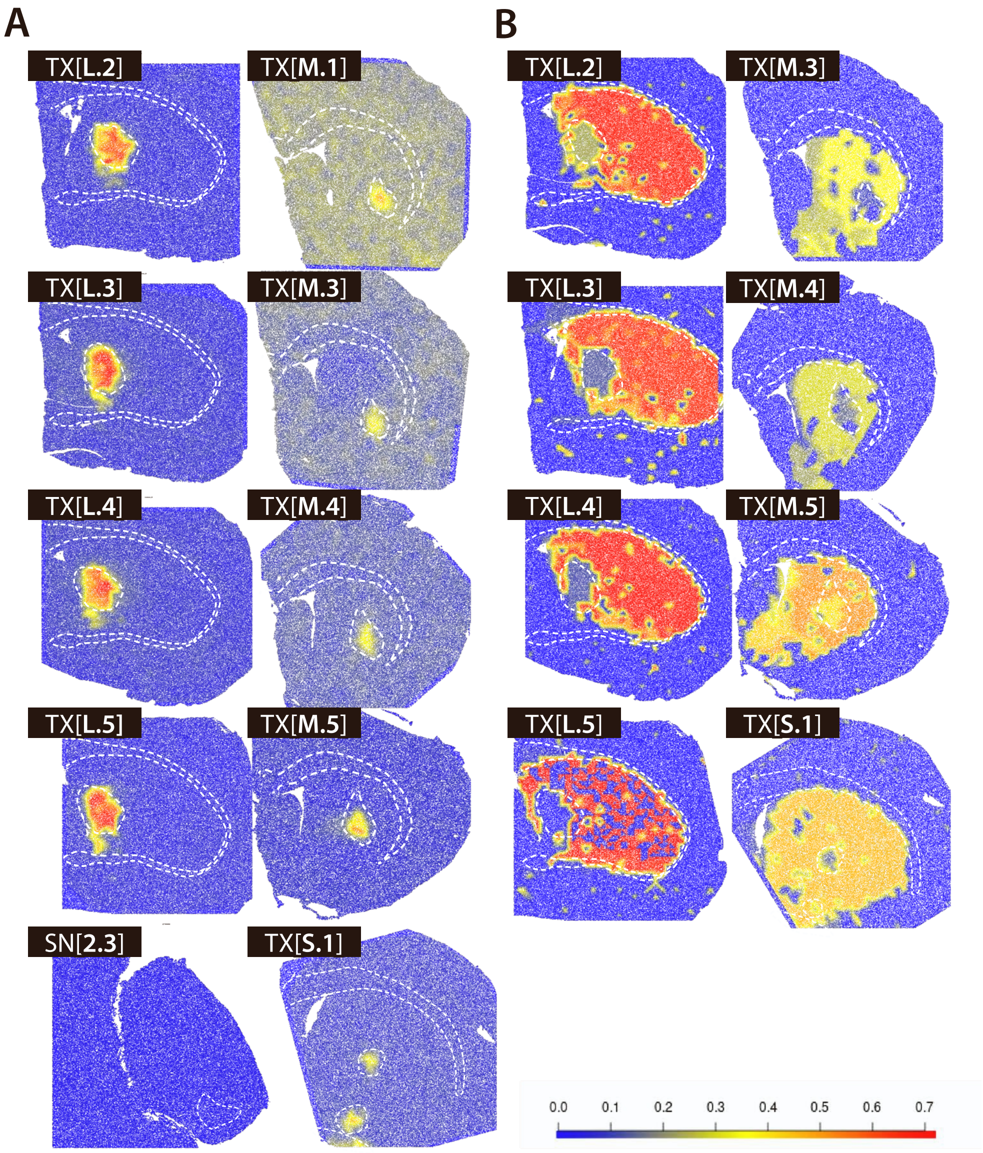

Figure S3: (a) Proportion of transcripts aligning to a unique site on the human genome in grafted striatal tissues. (b) Striatal score for forebrain tissue sections as assigned by cumulative expression of PENK, ADORA2A and PPP1R1B MSN marker genes.

Figure S4

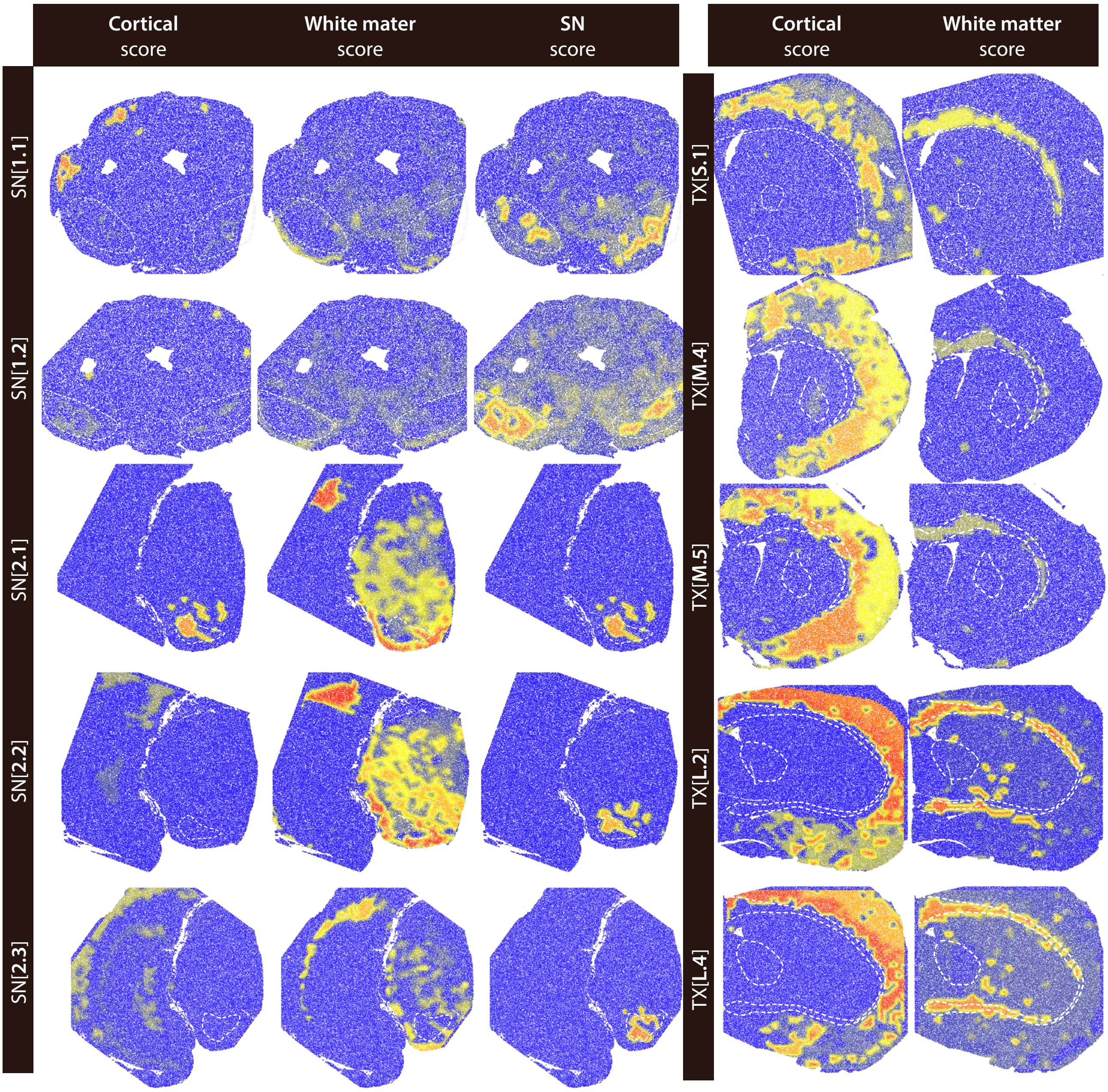

Figure S4: Region assignment through cumulative marker gene expression scores. Shown scores of Seurat-inferred clusters for expression of cortical, white matter or substantia nigra related genes in midbrain (SN[1-3]) and forebrain (TX[S-L]) sections. Dashed white guidelines are provided as guidelines for substantia nigra regions in midbrain sections and white matter tracts and grafted areas in forebrain sections.

Figure S5

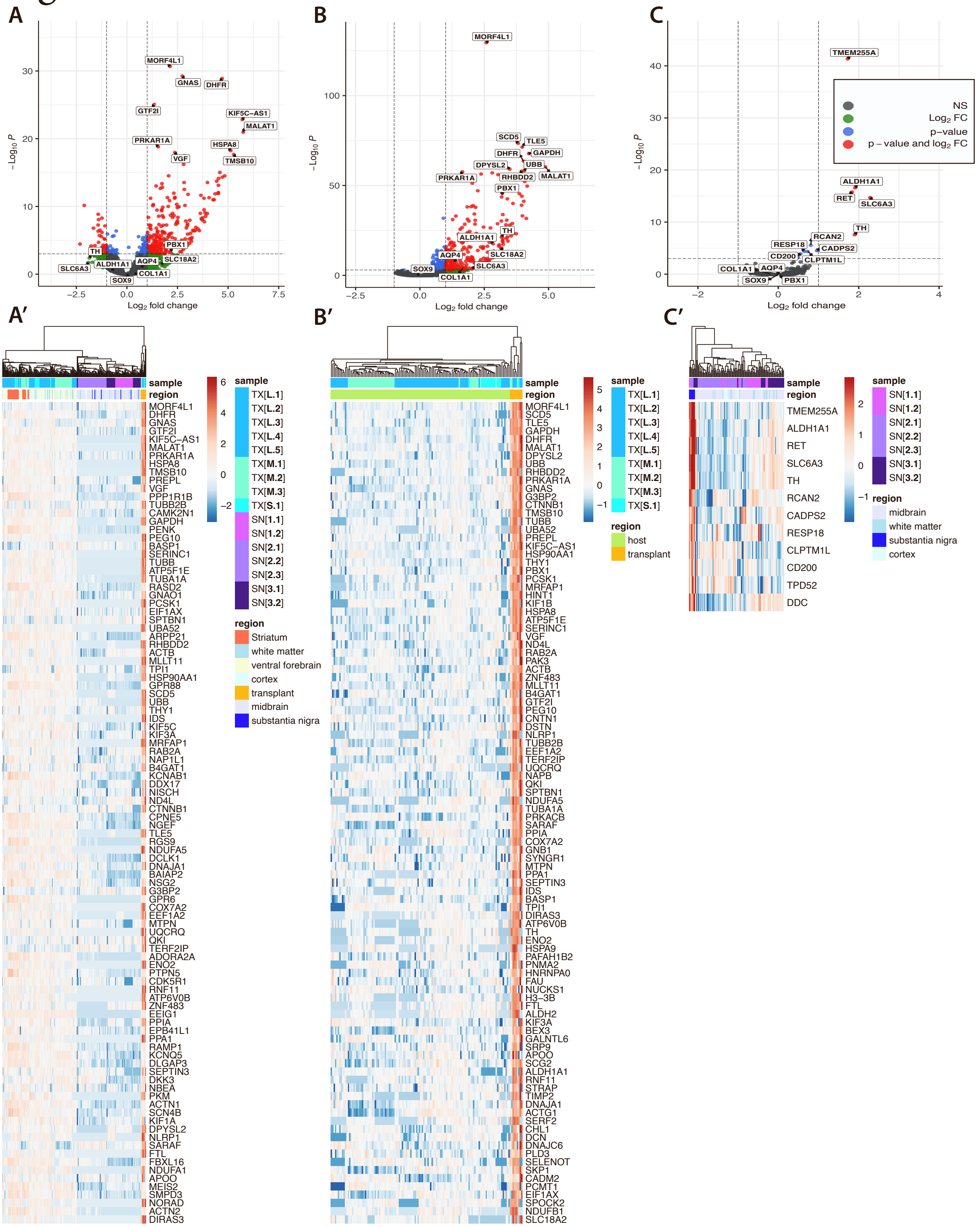

Figure S5: (a) Differentially expressed genes between the transplant and SN regions. Shown 10 genes with highest log2fold score and marker genes for dopaminergic neurons, VLMCs and mature astrocytes. (a') Top 100 genes with the highest log2fold change between the transplant and substantia nigra. Their scaled expression in other tissues is shown for comparison. (b) Differentially expressed genes between host tissues in the grafted forebrain regions and the transplants. (b') Top 100 genes with the highest log2fold change between the transplant and the remaining host tissue in the grafted forebrain sections. (c) Differentially expressed genes between the SN and the surrounding midbrain tissue. (c') Top 100 genes with the highest log2fold change between the SN and the surrounding midbrain tissue.

Figure S6

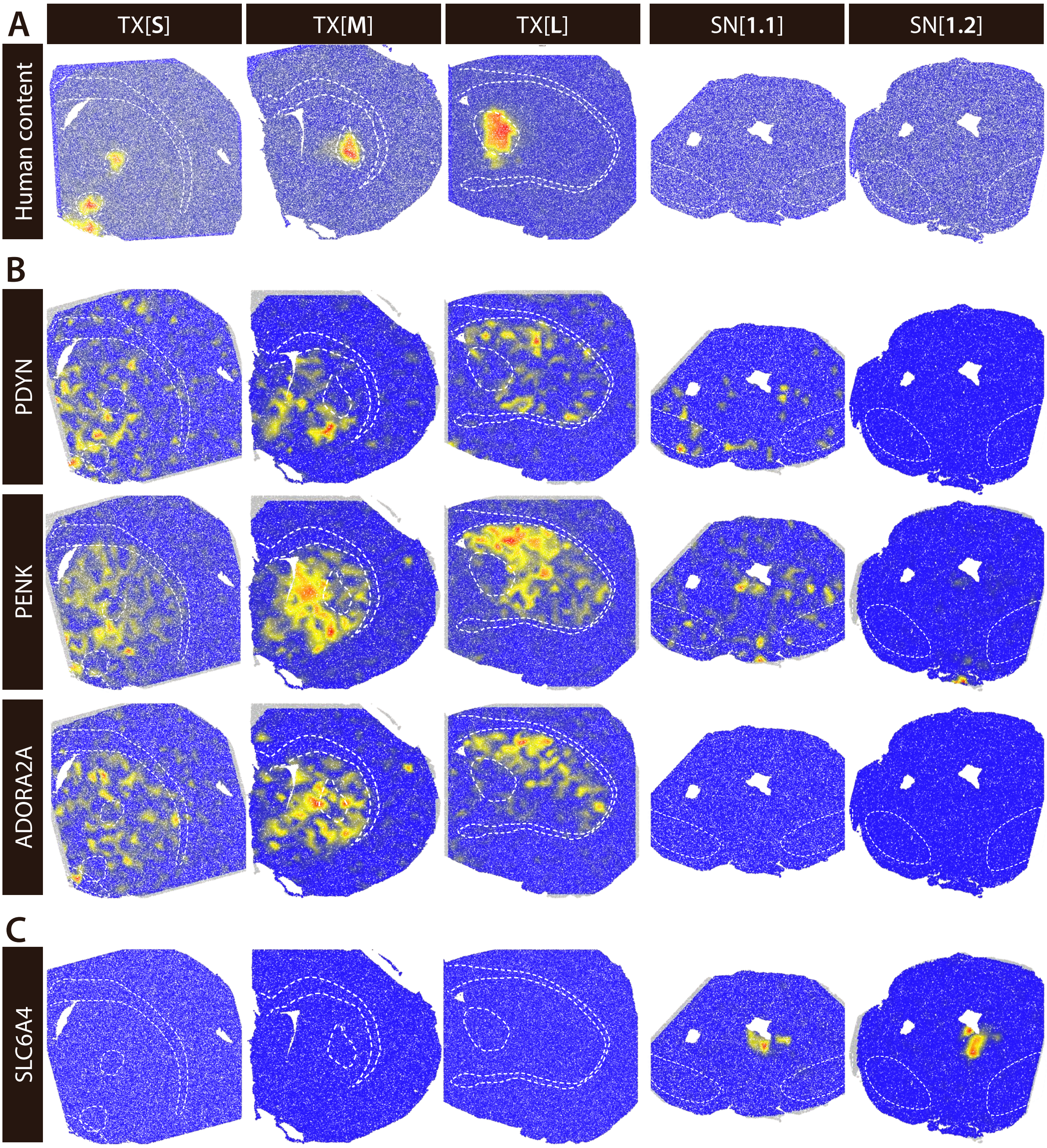

Figure S6: (a) Comparison of human transcript-rich areas in the grafted tissues and the human-assigned transcript content in the control midbrain tissues. (b) Striatal markers PDYN, PENK and ADORA2A expression in striatal tissues compared to the midbrain sections. (c) The expression of serotonin receptor SLC6A4 in the transplanted sections as compared to the midbrain sections, where SLC6A4 is present in the periaquedactal gray area. Dashed white guidelines are provided as guidelines for substantia nigra (SN) regions in midbrain sections and white matter tracts and grafted areas in forebrain sections.

Figure S7

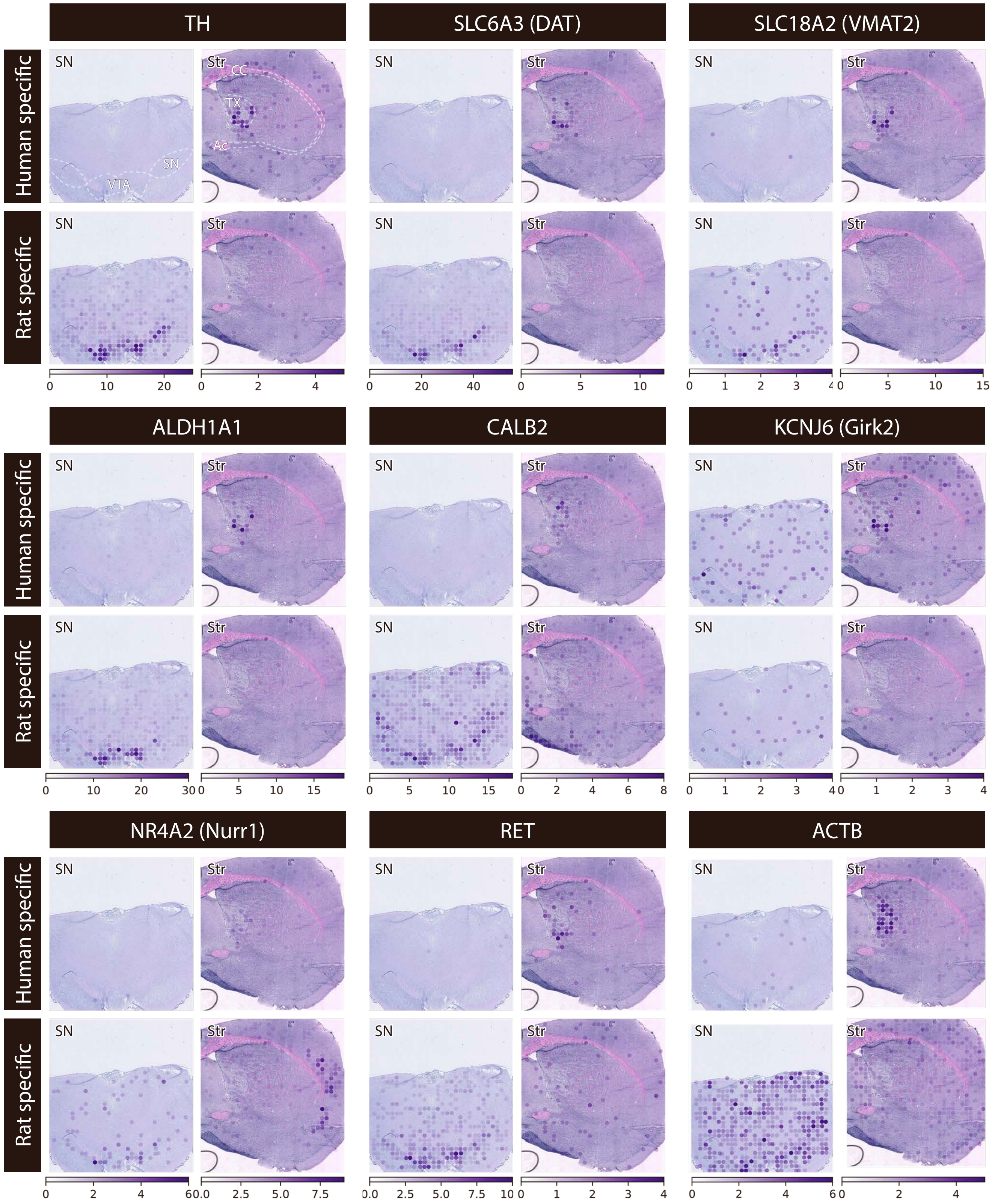

Figure S7: Expression of species specific transcripts for DA neuron marker genes and beta-actin (ACTB) in a rat SN section and grafted striatal section. VTA - ventral tegmental area, SN - substantia nigra, CC - corpus callosum, TX - transplant, Ac - anterior commisure.

Figure S8

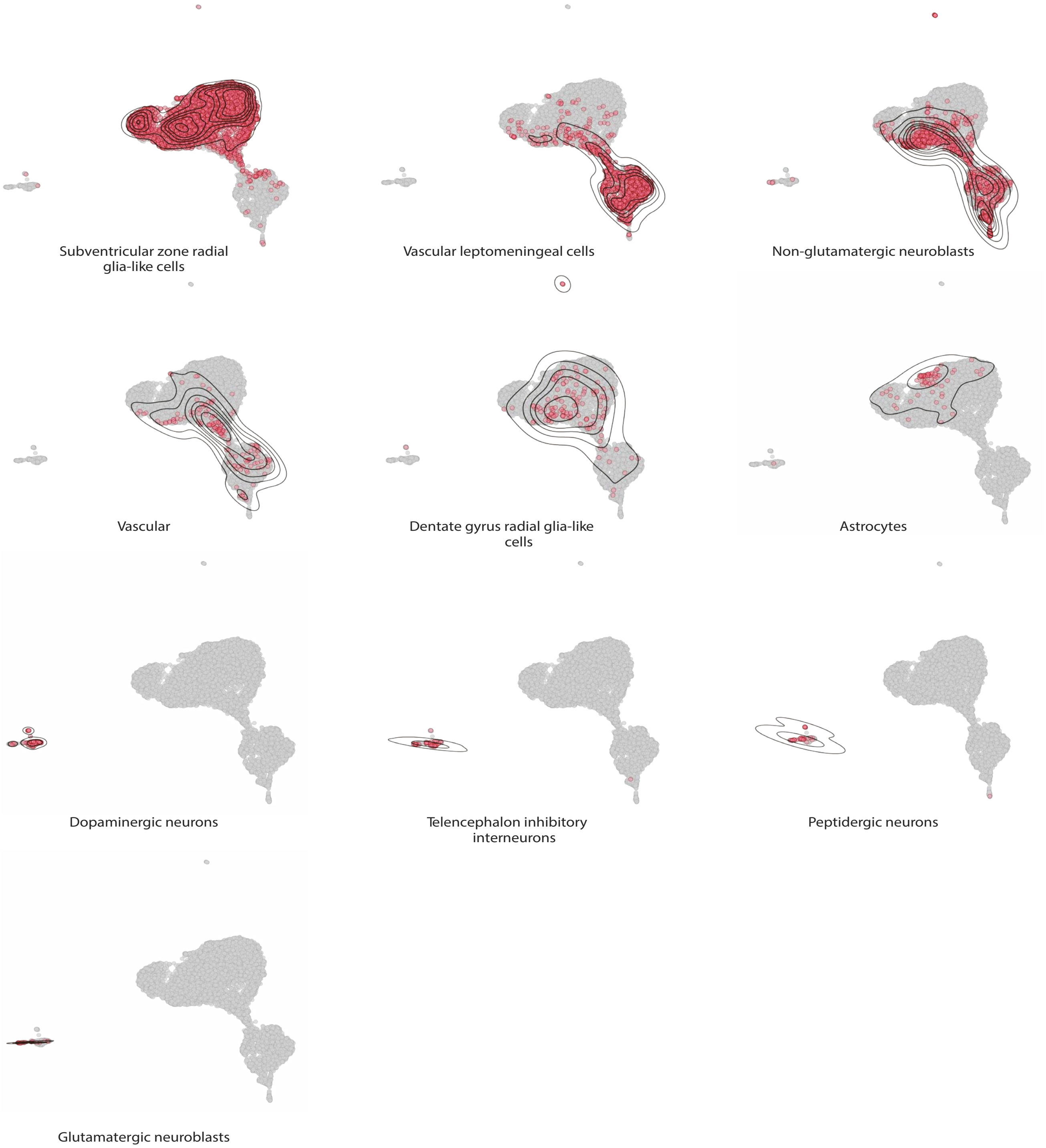

Figure S8: SingleR assignment of cells from a dopaminergic transplant using scRNAseq analysis. Presented are all cell types that were assigned more than 10 cells ordered from the most to least abundant. Contour plots show the density of cells assigned to UMAP space.

Figure S9

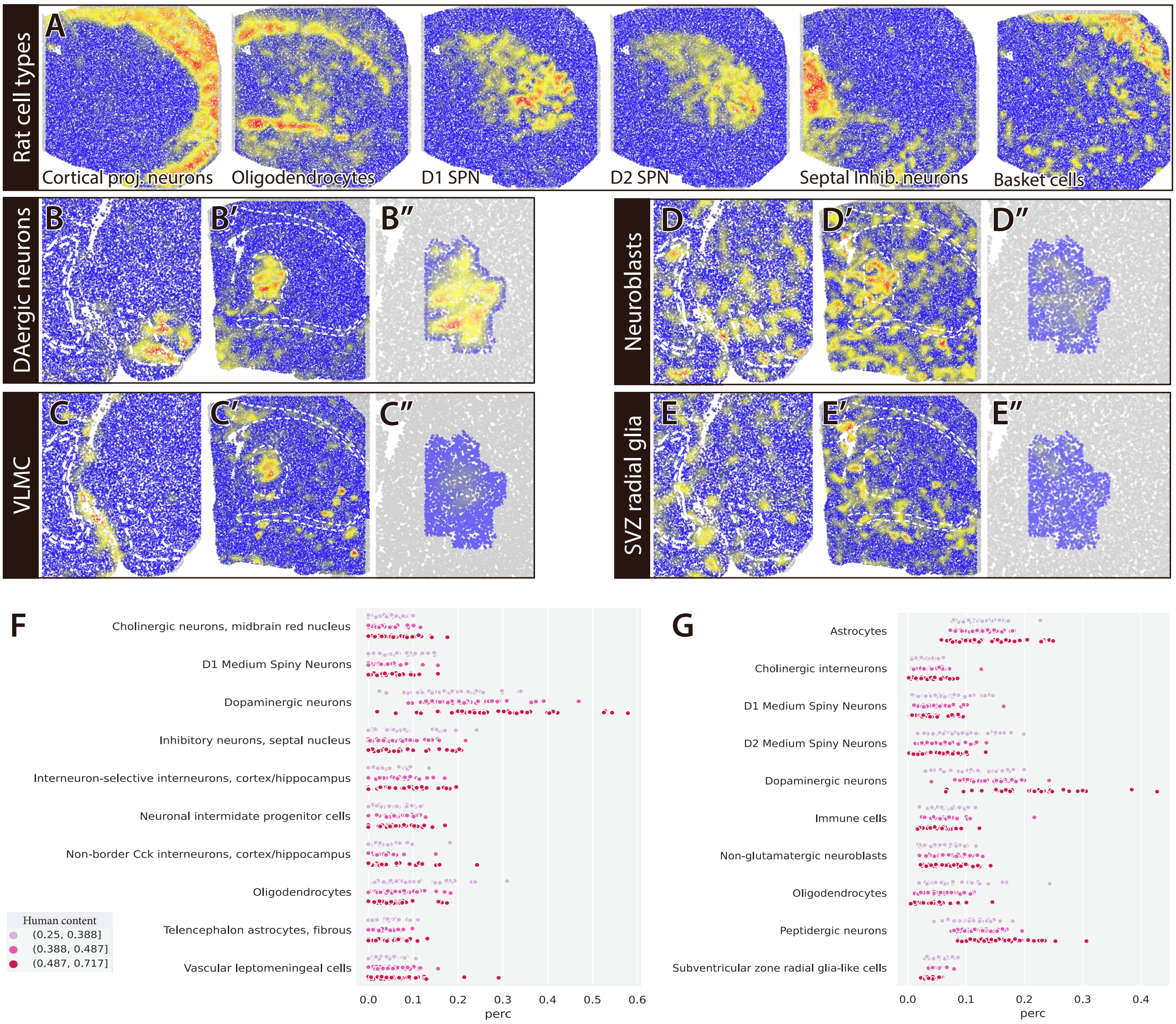

Figure S9: (a-e”) Deconvolution of grafted striatal and midbrain tissue sections with stereoscope. (a) Presence of host cell types. (b-e”) Presence of cell types previously observed in hESC-derived dopaminergic transplants. Shown is deconvolved cell type representation in midbrain sections with highlighted substantia nigra region (b-e), grafted striatal tissues (b’-e’) and only the transplant area (b”-e”). (f) Representation of cell types in features with increasing proportions of human reads as analyzed with stereoscope. (g) Representation of cell types in features with increasing proportions of human reads as analyzed with cell2location.

Figure S10

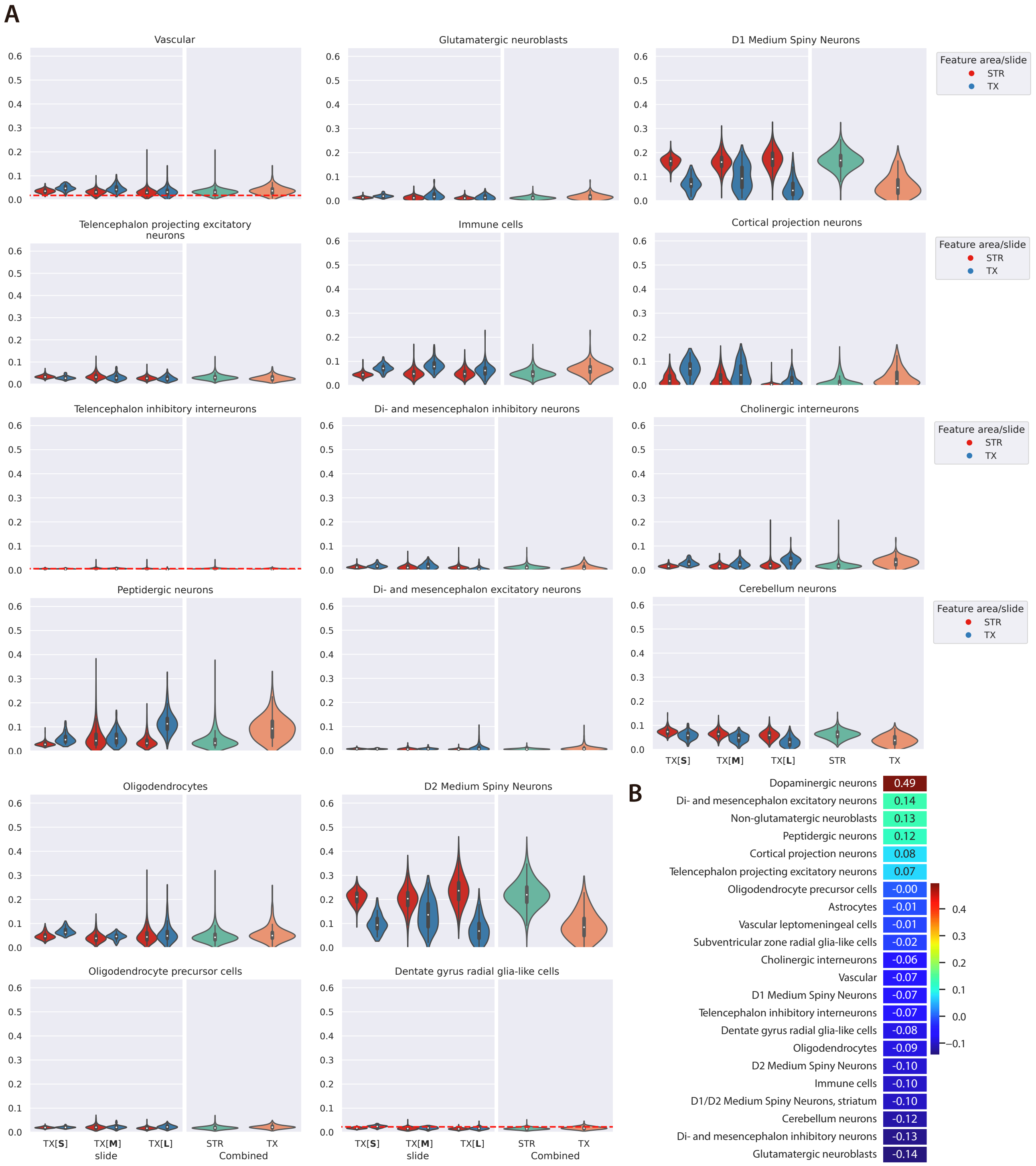

Figure S10: (a) Estimated proportions of reference dataset cell types in striatal and TX assigned features. Red line signifies cell type proportion of a cell type assigned to more than 10 cells scRNAseq analysis of hESC dopaminergic transplants as assigned by SingleR. (b) Correlations of proportions of human transcripts in a feature to a cell type content.

Figure S11

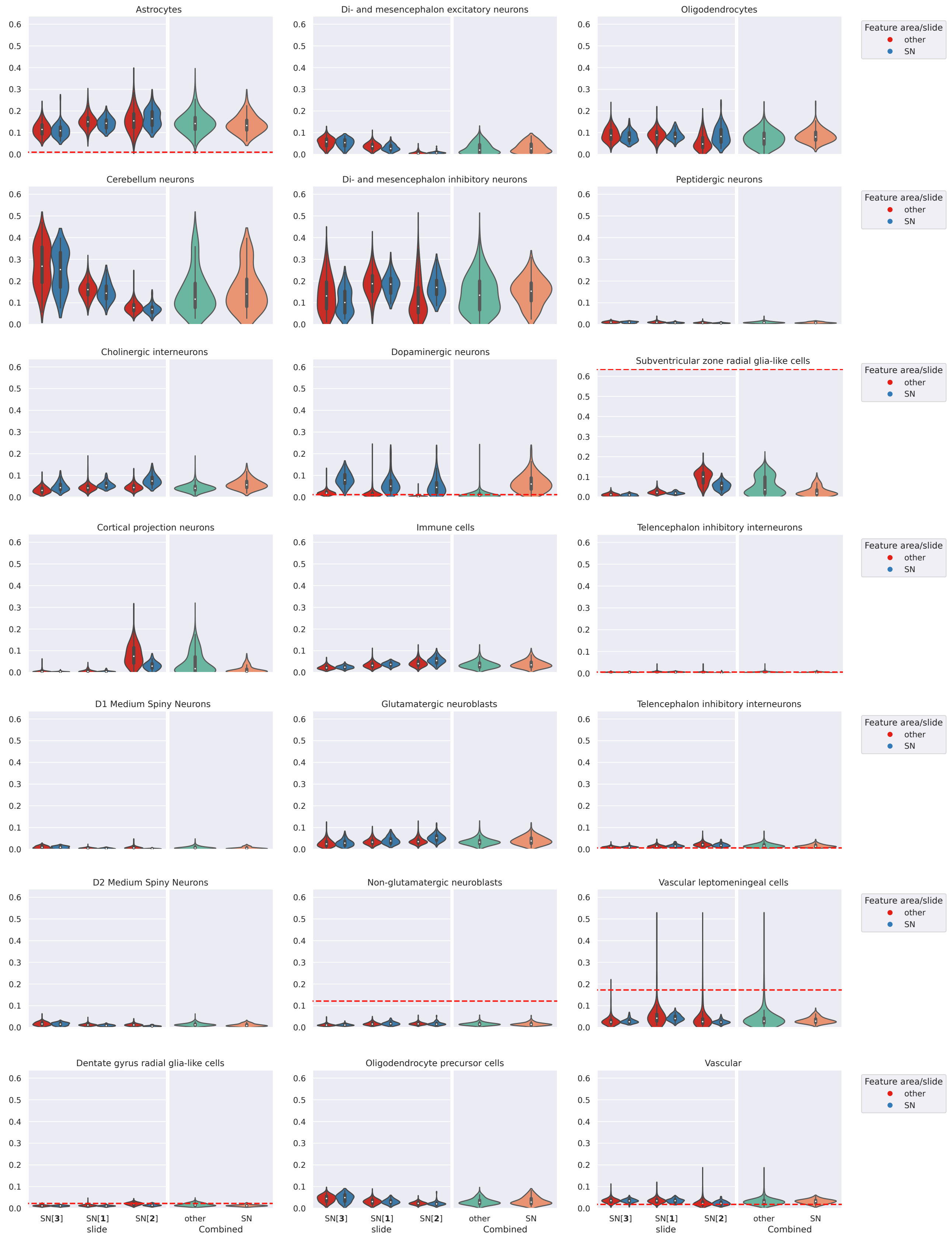

Figure S11: Proportion of cell types in substantia nigra as compared to the remainder of midbrain regions. Red line corresponds to the proportion of a cell type in scRNAseq analysis of dopaminergic transplants as assigned by SingleR

Figure S12

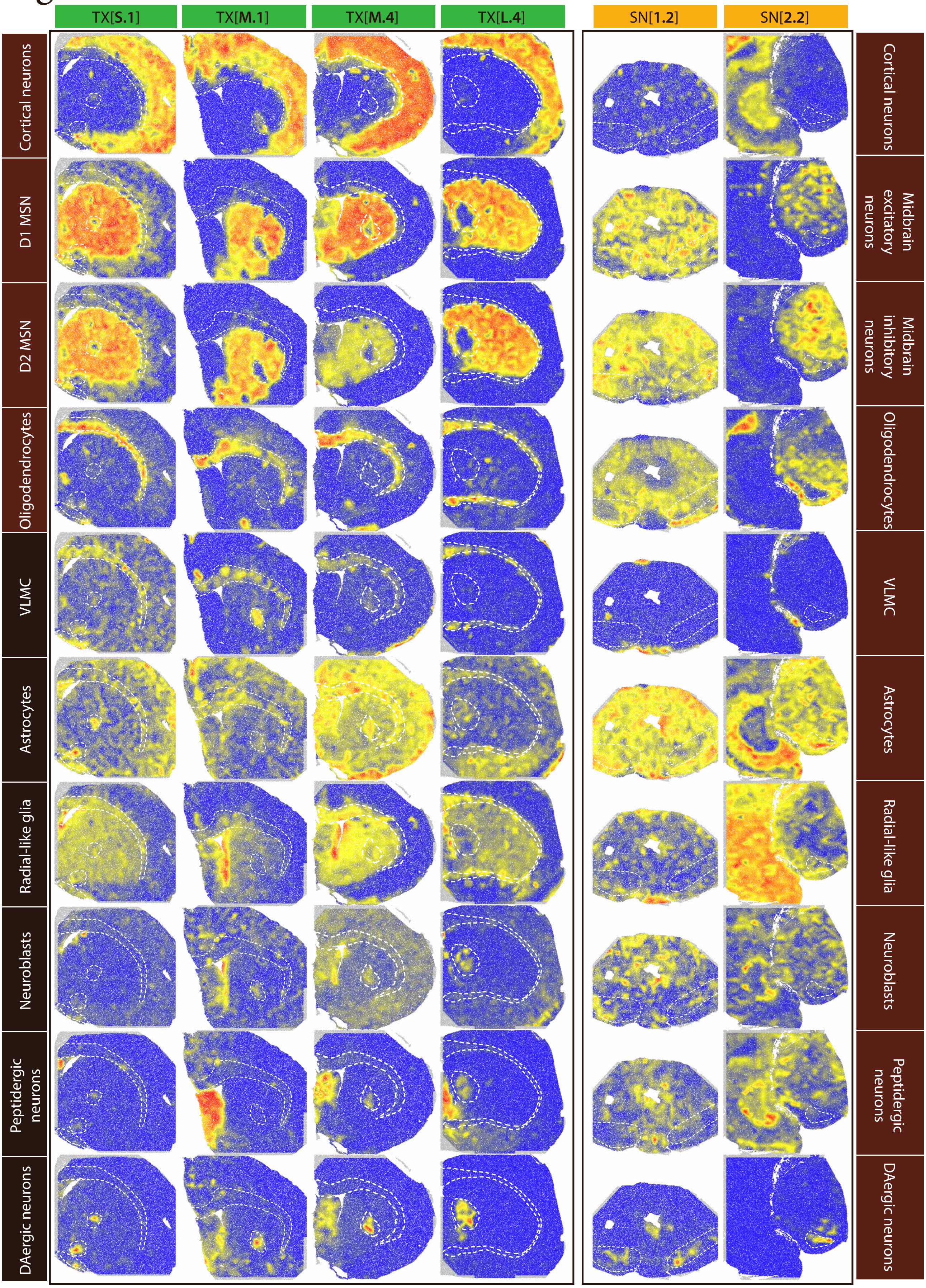

Figure S12: Relative distribution of cell types in representative transplanted sections versus in midbrain sections with substantia nigra region. Host cell types are marked with red and cells found in human transplants with black background. Dashed white guidelines are provided as guidelines for substantia nigra regions in midbrain sections and white matter tracts and grafted areas in forebrain sections.

Figure S13

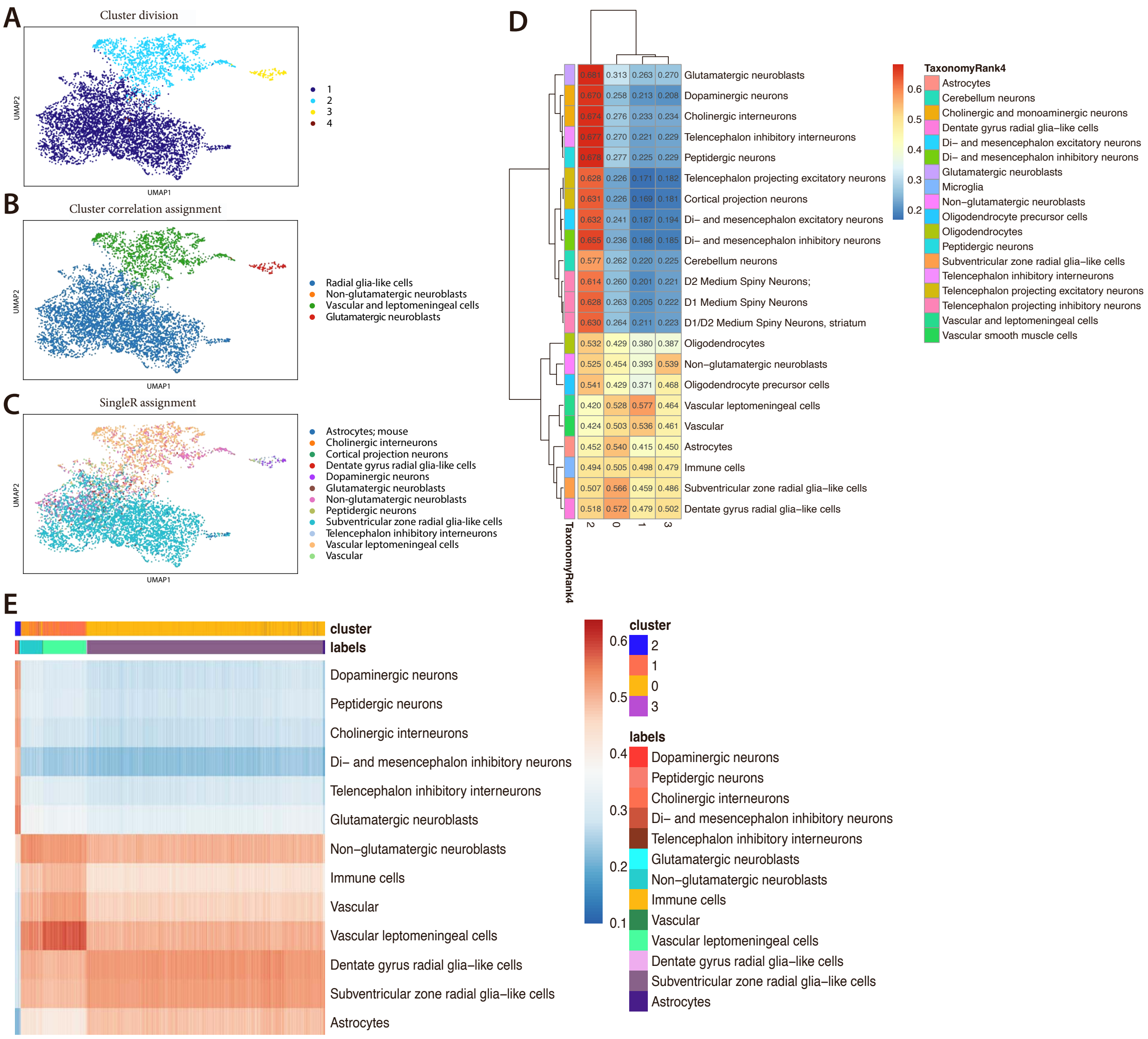

Figure S13: (a-c) UMAP representation of hESC derived dopaminergic graft scRNAseq analysis annotated by (a) cluster assignment, (b) correlation of clusters with the reference dataset cell types and (c) SingleR assigned cell types using the same reference dataset as in (d) and (e). (d) Spearmann correlation of clusters' transcriptome with that of the reference dataset cell types'. (e) All cell type scores for SingleR annotation of hESC derived dopaminergic graft scRNAseq analysis.
