# Supplementary material for: Deconvolution of spatial sequencing provides accurate characterization of hESC-derived DA transplants in vivo
Source: Mol Ther Methods Clin Dev. 2023 May 4;29:381–94. doi: 10.1016/j.omtm.2023.04.008 (PMC10209706; doi:10.1016/j.omtm.2023.04.008)
Supplement: Document S2. Article plus supplemental information [file mmc2.pdf]

Molecular Therapy
Methods & Clinical Development
Original Article

Deconvolution of spatial sequencing provides accurate characterization of hESC-derived DA transplants *in vivo*

Jana Rájová,[1] Marcus Davidsson,[1] Martino Avallone,[1] Morgan Hartnor,[1] Patrick Aldrin-Kirk,[1] Tiago Cardoso,[2] Sara Nolbrant,[2] Annelie Mollbrink,[3] Petter Storm,[2] Andreas Heuer,[4] Malin Parmar,[2] and Tomas Björklund[1]

[1]Molecular Neuromodulation, Department of Experimental Medical Science, Lund University, 221 84 Lund, Sweden; [2]Developmental and Regenerative Neurobiology, Department of Experimental Medical Science, Lund University, 221 84 Lund, Sweden; [3]Science for Life Laboratory, Division of Gene Technology, KTH Royal Institute of Technology, 106 91 Stockholm, Sweden; [4]Behavioural Neuroscience Laboratory, Department of Experimental Medical Sciences, Lund University, 221 84 Lund, Sweden

Cell therapy for Parkinson's disease has experienced substantial growth in the past decades with several ongoing clinical trials. Despite increasing refinement of differentiation protocols and standardization of the transplanted neural precursors, the transcriptomic analysis of cells in the transplant after its full maturation *in vivo* has not been thoroughly investigated. Here, we present spatial transcriptomics analysis of fully differentiated grafts in their host tissue. Unlike earlier transcriptomics analyses using single-cell technologies, we observe that cells derived from human embryonic stem cells (hESCs) in the grafts adopt mature dopaminergic signatures. We show that the presence of phenotypic dopaminergic genes, which were found to be differentially expressed in the transplants, is concentrated toward the edges of the grafts, in agreement with the immunohistochemical analyses. Deconvolution shows dopamine neurons being the dominating cell type in many features beneath the graft area. These findings further support the preferred environmental niche of TH-positive cells and confirm their dopaminergic phenotype through the presence of multiple dopaminergic markers.

INTRODUCTION

The degeneration of dopaminergic neurons in Parkinson's disease (PD) is the leading cause of motor, and some non-motor, symptoms of the disease.[1] This has resulted in the development of therapies attempting to re-supply the lost dopamine (DA) signaling in the brain, ranging from pharmacological interventions[2,3] to cell-based therapies.[4–6] Cell-based therapies placing DA-producing grafts in the putamen[7,8] entered the scene several decades ago.[9–12] Initially, the dopaminergic neuroblasts were isolated from the early ventral mesencephalon (VM) derived from human fetal tissue.[7,13] However, because of the fundamental issues with this approach,[14] research has moved toward the differentiation of VM dopaminergic neurons from pluripotent stem cells.[15,16] Human embryonic stem cells (hESCs) are the most researched, replenishable source of dopaminergic transplants[17–20] although human induced pluripotent stem cells (hIPSCs) have also been used successfully.[21,22]

Many studies have shown the efficiency of DA transplants in ameliorating motor function in animal models of PD,[23–28] leading to the approach entering clinical trials.[5,29,30] New clinical studies based on hESC-derived DA progenitors have recently been initiated in the United States and Sweden (clinicaltrials.gov: NCT04802733 and clinicaltrials.gov: NCT05635409). Long-term follow-up of fetal transplants has shown their potential for alleviating motor symptoms in PD patients for nearly two decades after transplantation[7,31] without additional treatment.[7]

As mature dopaminergic neurons will not survive grafting, dopaminergic neural VM progenitors, which are not fully maturated, are used for transplantation.[32–34] This approach, however, leaves the transplant's final composition challenging to predict and control.[35] The final graft composition *in vivo* from hESC-derived dopaminergic transplants has been analyzed in bulk[36] and at single-cell resolution.[18] However, the quantitative results of the latter study were at odds with histological assessments since the sequencing study suggested that neurons make up the minority of the transplant. When assessed *in situ* with immunohistochemistry (IHC), cells positive for neuronal and dopaminergic markers represent over 50% of all cells in a transplant.[18,37] As the authors suggest,[36] this discrepancy could result from the cells' differential sensitivity to the processing as the cell proportions were skewed toward the more robust non-neuronal cells. Differences in cell composition across RNA sequencing technologies is a well-known phenomenon,[36,38] further supporting the need for minimal processing of the samples for unbiased results.

To overcome cell type specific biases and gain more insight into the transplants in the context of their environment, we used the spatial transcriptomics (ST) workflow.[39] We analyzed, in depth, three hESC-derived dopaminergic transplants matured and integrated

Received 9 January 2023; accepted 28 April 2023;
https://doi.org/10.1016/j.omtm.2023.04.008.

Correspondence: Tomas Björklund, Molecular Neuromodulation, Lund University, BMC, A10 22184 Lund, Sweden.
E-mail: tomas.bjorklund@med.lu.se

© 2023 The Author(s).
This is an open access article under the CC BY license (http://creativecommons.org/licenses/by/4.0/).

within the host tissue. Among the advantages of ST analysis are the preservation of the spatial relationships and minimal tissue preprocessing, which favors an accurate snapshot of the tissue state. Spatial methods are developing in several directions. Some sacrifice single-cell resolution in favor of unbiased transcriptome capture,[39–41] while others focus on high resolution and detection of predetermined genes.[42–45]

Although spatial data can give crucial information about the analyzed tissue, most currently available methods are not standardized enough to be used in clinical development while retaining the ability to capture the entire transcriptome. With commercialization, ST has overcome much of this hurdle and it has also been shown to produce valuable data in clinical research.[46–49] As hESC-based transplantation is now entering clinical trials in PD, we have chosen to generate the data for those cells on the basis of ST as a foundation for future post-mortem analysis.

RESULTS

Generation of dopaminergic transplants matured in an animal model

In this study, dopaminergic progenitors, derived from hESCs and differentiated *in vitro* toward a VM phenotype, were grafted into the lesioned striatum of a toxicant-induced immunocompromised (nude) rat model of PD (Figure 1A). 6-hydroxy-DA (6-OHDA) was injected into the right medial forebrain bundle resulting in a near-complete unilateral depletion of dopaminergic neurons in the substantia nigra pars compacta (SNpc).

The animals were sacrificed 6–15 months after grafting to allow functional maturation of the grafted cells. Brain tissue was flash-frozen and cryosectioned, coronal sections were placed on amine-binding slides (Surmodics) with ST oligo-dT array-printed spots (100μm in diameter).[39] After fixation, tissue was stained with H&E, revealing grafted areas in the injected striatal region (Figures 1B–1B″).

The mRNA from the striatal tissue and the SNpc control sections was then subjected to the ST protocol.[50] Briefly, the RNA is hybridized to the barcoded oligo-dT probes on the glass surface, the cDNA is generated *in situ*, and the tissue is digested. After that, the cDNA is cleaved away from the glass, linearly amplified, and prepared for Illumina sequencing. With the help of the coordinate-specific, barcoded probes printed in the array spots, the transcriptomic profile of each spot can then be projected back onto the tissue. The number of transcripts per feature for all samples is shown in Figure S1. As there are transcripts of two species being analyzed, the transcripts have been aligned to a chimeric rat and human genome. For gene expression analysis, the species identity was stripped away. However, transcripts aligning uniquely to a single species give a good glimpse into the species composition of features.

Batch effect detection and removal

Because of the complexity of the procedure, we first assessed the animal-to-animal and section-to-section variability to see the similarity between biological replicates (Figures 1C and C′). Without performing any batch correction, joint analysis of tissues from close anatomical sites showed considerable variation, both among sections and animals, with no meaningful overlap between sections. These differences among such biologically close tissues pointed to nested batch effects. To correct for these effects, we compared two emerging batch correction approaches, Scanorama[51] (Figures 1D and D′) and DESC[52] (Figures 1E and E′), to the commonly used batch correction algorithm within Seurat[53] (Figures 1F and 1F′). Although aimed primarily at single-cell RNA sequencing (scRNA-seq) datasets, most of these have previously been applied to ST data as well, with Seurat being specifically incorporated into the spatial data-specific module STUtility.[54]

Although all three algorithms significantly diminished the distance among biological replicates, there were visible differences in how clusters based on the corrected gene expression were separated. All algorithms separated striatal, cortical, and septal regions, the white matter tracts, and the transplanted areas (Figures 1C′–1F′) with varying degrees of modularity. Whereas DESC and Seurat managed to separate tissues on the basis of morphological areas, Scanorama showed a level of separation based on the host animal (Figures 1D and 1D′). Notably, unlike most anatomical structures, grafts were united throughout all animals, indicating a higher degree of similarity among them.

DESC, on the other hand, fully integrated all the sections regardless of the host. However, besides a handful of major clusters connected to morphological areas, about twice as many small communities appeared, not clearly related to anatomical regions (Figures 1E and 1E′).

Seurat was best able to correct for batch effects both on the level of animals and sections while not sacrificing division within the tissues themselves or creating outlier clusters (Figures 1F, 1F′, and S2), as seen with DESC. The uniform manifold approximation and projection (UMAP) representation of the corrected dataset does not point to a bias at either slide, animal, or tissue section level. There is also an apparent separation of layers and functional regions within the cortex, in line with what is expected from the known anatomy of the rat brain.[55] Taken together, these findings support further continuation with the Seurat algorithm clustering.

Identification of human tissue on the transcriptome level

To properly delineate the grafted and host areas (Figures 2A–2A″), we classified features on the basis of the ratio of transcripts aligning solely to a single location on either the human or the rat genome. In line with the expectations, areas with a high percentage of human transcripts were observed in regions with a histologically visible transplant (Figures 2B–2B″ and S3A). Outside of these regions, the fraction of human reads tends to be negligible and can serve as a proxy for the quality of the well processing. High signal from human reads uniformly distributed throughout tissues can point to an ST sample with template switching caused by over-amplification and can be used to exclude sections with low signal-to-noise ratios.

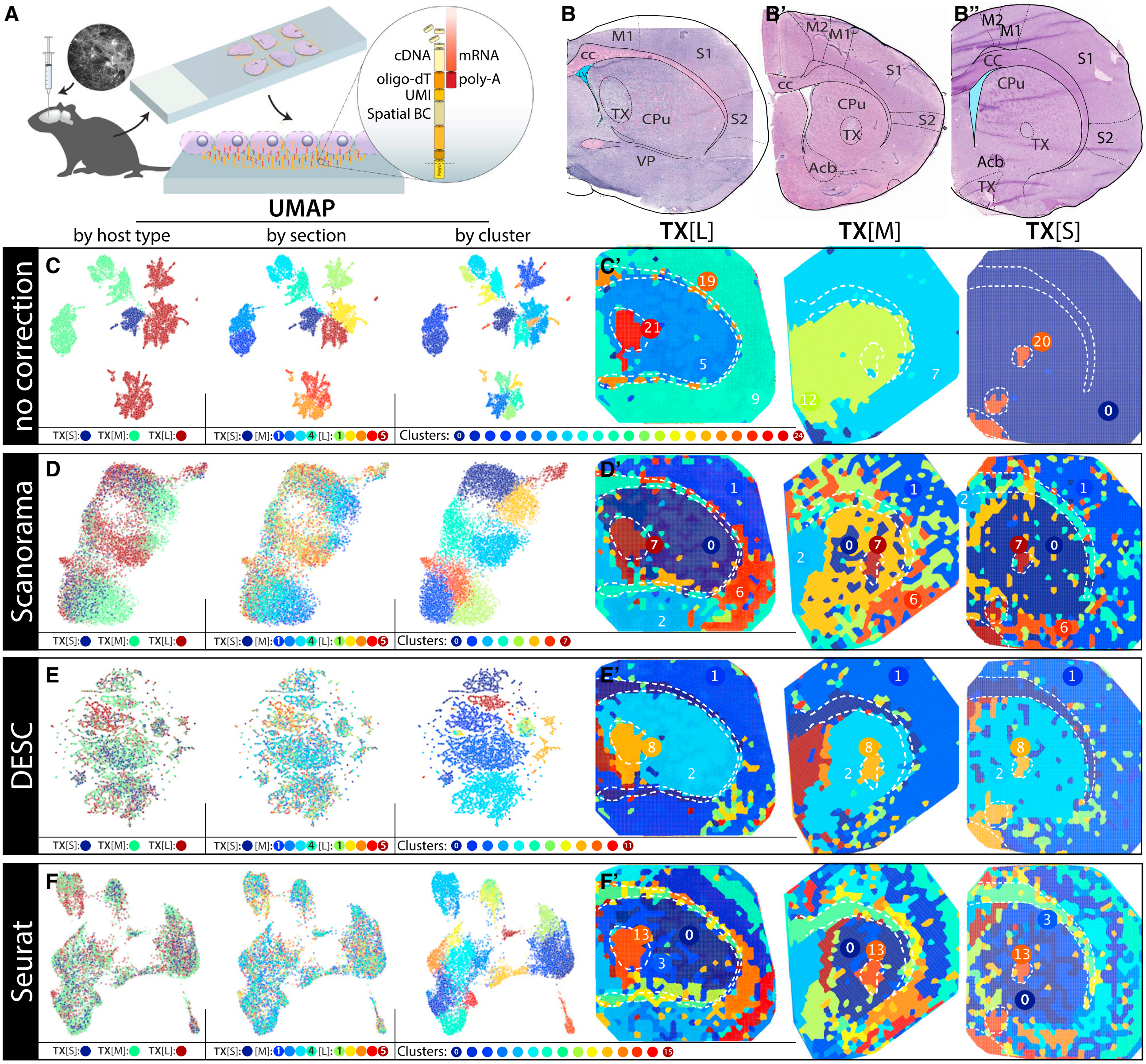

Figure 1. Comparison of batch correction approaches for Spatial Transcriptomics

(A) Schematic of ST slides and the mRNA capturing probes immobilized on the features. (B–B″) H&E stain of sections with large (B), medium (B′), and small (B″) transplant in forebrain tissue. TX, transplant; CPu, caudate putamen; M1/M2, primary/secondary motor cortex; S1/S2, primary/secondary somatosensory cortex; cc, corpus callosum; Acb, nucleus accumbens; VP, ventral pallidum. (C–F′) ST dataset of all transplanted sections. (C) UMAP of the ST dataset, labeled by the host, section, or cluster. (C′) Clusters' distribution throughout the tissue sections. (D–F′) UMAP with Scanorama (D and D′), DESC (E and E′), and Seurat (F and F′) batch corrected dataset and the distribution of clusters in tissues.

Identification of anatomical areas

The Seurat corrected data were used as a basis for differential gene expression analysis aimed at distinguishing transcripts specific to the transplants (Figures 2A–2A″). The transplant area was defined as a Seurat cluster with the highest human content. Because of the placements of the grafts, we have selected the surrounding striatum as the contrast region. The host striatum has been selected through feature clusters rich in medium spiny neuron (MSN) marker genes,[56] specifically *PENK*, *ADORA2A*, and *PPP1R1B*. This cluster outlined the striatal area as expected on the basis of the morphology of the tissue. In all cases, the expression of MSN marker genes strongly diminished in the transplant areas, confirming the differing composition of the transplant to its surrounding tissue (Figures 2C–2C″ and S3B). Furthermore, SNpc, white matter tracts, and cortical areas were

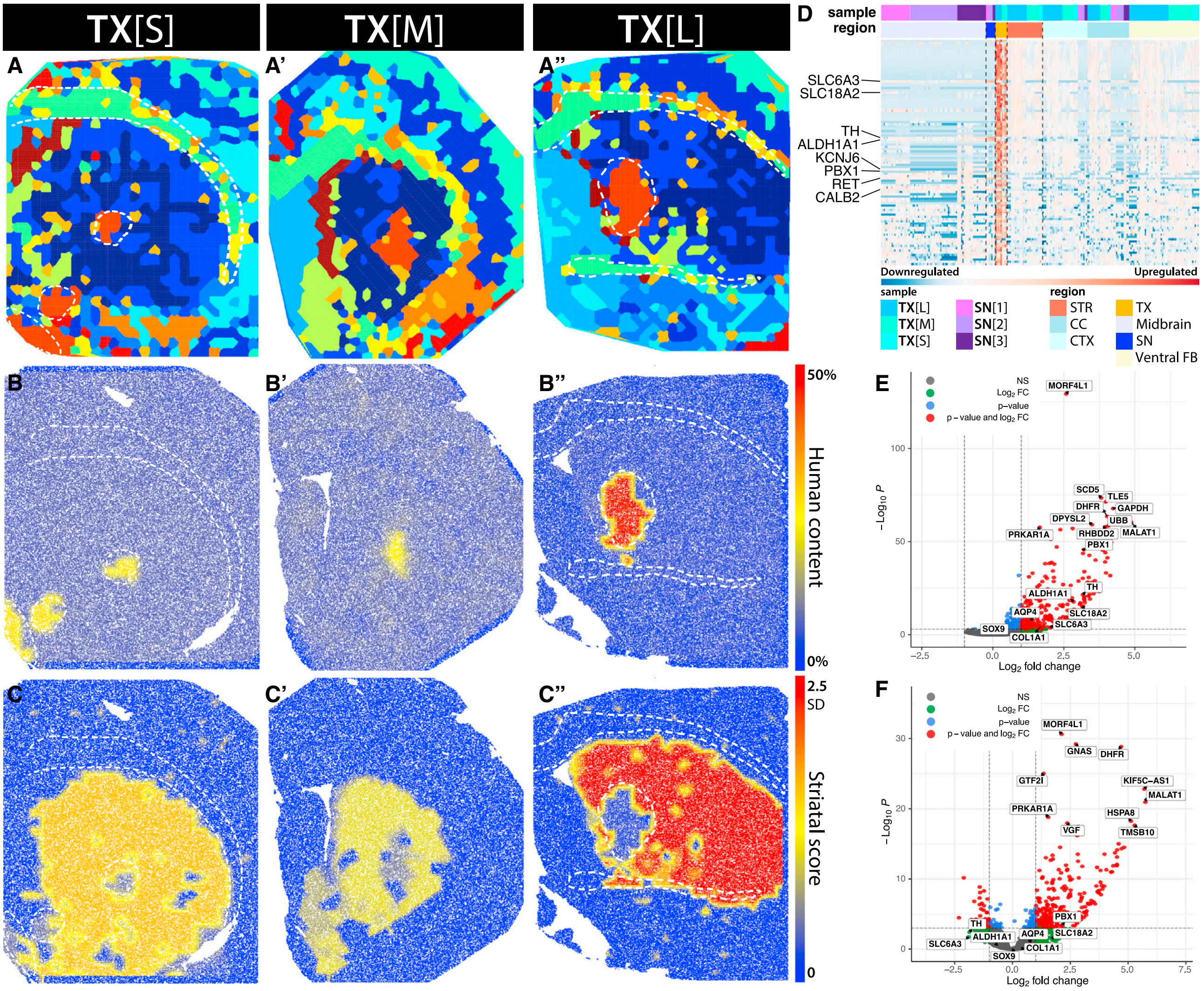

Figure 2. Assessment of human content and differentially expressed genes in the hESC-derived transplants

(A–A″) Seurat-assigned clusters in tissues with transplants and their respective score for (B–B″) human transcript content and (C–C″) MSN-associated gene content. (D) Heatmap of genes most differentially expressed between striatum and transplant regions and the expression of these genes in the remaining regions found in the ST processed tissues. (E) Genes differentially expressed between the transplants and the remainder of the grafted tissues. (F) Differentially expressed genes between the substantia nigra and the transplants.

defined by their own marker genes (materials and methods; Figure S4). The transplants have been defined as clusters comprising more than 15% human transcripts.

To overcome variability on the level of unique features for differential gene expression, all regions of interest within a tissue were treated as a pseudobulk to support the robustness of the analysis.

Differential gene expression was assessed by modeling the data on the basis of negative binomial distribution as implemented in the DESeq2 statistical module. We assessed transcriptomic differences in three scenarios: Between the transplant and the striatum, the transplant, and all the remaining host tissue in the grafted sections, and the transplant and the SNpc area (Figures 2D–2F, S5A, and S5B′). When looking at the genes most differentially expressed in the transplant compared with the striatum, we can see many well-established dopaminergic markers (Figure 2D). Although these markers are very sparsely represented in the remainder of the grafted host tissues, they can be seen highly expressed also in the SNpc, pointing to a shared robust dopaminergic population. Many of these genes are also highly differentially expressed in the SNpc compared with the rest of the midbrain, cortical, and white matter regions, supporting the uniqueness of the dopaminergic population in both regions (Figure S5C). These differences are also recapitulated in the analysis between the transplant areas

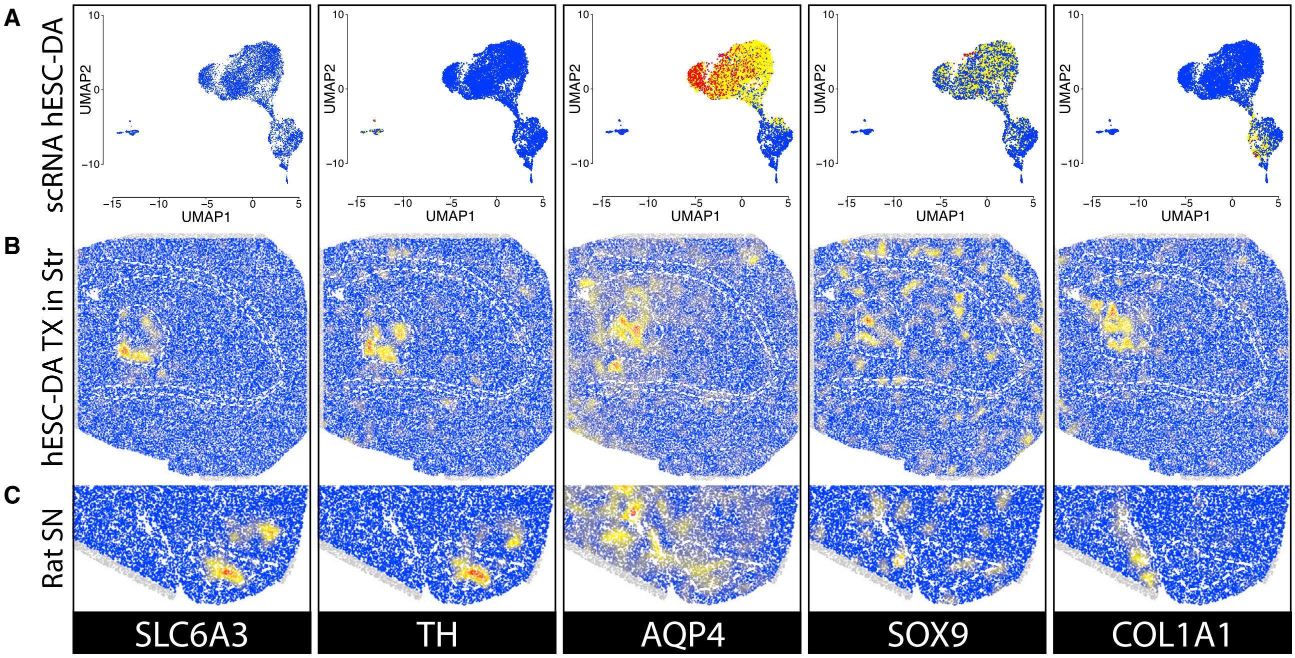

Figure 3. Comparison of gene expression in a scRNA-seq and Spatial Transcriptomics analysis of hESC-derived DA neurons

Expression of genes associated with dopaminergic neurons (SLC6A3, TH), astrocytes (AQP4, SOX9), and VLMCs (COL1A1) in (A) scRNA-seq of previously analyzed transplant, (B) ST analysis of grafted tissue (transplant area is delineated in the medial striatum), and (C) ST analysis of intact substantia nigra.

and the surrounding host tissues (Figures 2E, S5B, and S5B′). Although some genes preferentially expressed in the grafts as opposed to the surrounding host tissue remain differentially expressed between the SNpc and transplants, dopaminergic markers are similarly expressed in both (Figures 2F, S5A, and S5A′).

Interestingly, there are very few or no significantly downregulated genes in the analysis of differentially expressed genes (DEGs) (Figures 2F, S5B, and S5C). This effect is most likely caused by a partial volume effect when features at the edges of unique areas are populated by cells from neighboring tissues. This is further supported by the results of a differential gene expression analysis between SNpc and the transplants, where classical MSN marker genes *PENK* and *PPP1R1B* show up as being expressed in the transplant. When assessing the expression of these genes on the ST assays, we can see a gradient of MSN markers from the striatum past the edges of the transplanted regions (Figure S6B).

ST versus scRNA-seq composition analysis

Dopaminergic transplants, generated using this hESC cell line (RC17) and differentiation protocol[37] have previously been deeply characterized using scRNA-seq.[20] In the reanalysis of this previous study, three major clusters were observed based on the transcriptomic profiles generated with the scRNA-seq data (Figures 3A and S13A). The smallest cluster carries neuronal characteristics, and the two larger clusters express glial and vascular leptomeningeal cell (VLMC) markers. The single-cell extraction protocol resulted in a very low recovery of neuronal cells, and only a small subset of these cells expressed canonical markers of dopaminergic neurons.[36] The neuronal population was, however, very well differentiated from the larger population of captured glial cells.

The presence of marker genes of dopaminergic neurons, astrocytes, and VLMC has been shown across the scRNA-seq data as well as in the ST analysis of both the grafted tissue sections and the non-lesioned substantia nigra regions (Figures 3B and 3C). Among dopaminergic neuron markers (*TH* and *DAT* [*SLC6A3*] shown), their expression concentrates toward the edges of the large transplant in the ST analysis while still covering a significant proportion of the transplants' features. Furthermore, species-specific analysis of the expressed DAergic marker genes in the grafted sections showed them to be of nearly universal human origin. In contrast, the opposite is true for the same marker gene transcripts in sections containing rat substantia nigra (Figure S7). In scRNA-seq, only a minority of the cells in the neuronal cluster show an expression of *TH* or *DAT*. Glial cell markers (*AQP4* and *SOX9* for astrocytes and *COL1A1* for VLMCs) are expressed mostly at the center of the graft *in situ* (Figure 3B).

Graft-induced dyskinesia observed from fetal grafts has been proposed to be due to the presence of serotonergic neurons in the grafts[14,57] or through an extrinsic serotonergic stimulation of the grafted DA neurons.[58] In the grafts differentiated with the current protocol, no serotonergic neuron markers were found in the ST analysis compared with markers found in the midbrain regions, pointing to an absence of a serotonergic population in the transplants at this stage of the maturation (Figure S6C).

Analysis of transplants' composition through scRNA-seq

To estimate cellular proportions in the dopaminergic grafts, we analyzed the scRNA-seq data using a reference dataset.[59] This dataset is composed of cell types from striatal, cortical, and ventral midbrain regions. Because of the imbalances in the dataset composition and the underrepresentation of cells positive for dopaminergic markers in the neuronal cluster, we chose SingleR, which assigns the probability of belonging to a cell type to each cell in the dataset separately. This approach avoids assigning cell identity on the cluster level and can resolve related populations such as the DA and other neurons. The SingleR assignment resulted in the cluster carrying neuronal characteristics being divided into multiple neuronal types, with dopaminergic neurons being the most prominent. Astrocytes, radial glial cells, VLMCs, and neuronal progenitors were distributed in a gradient throughout the two larger clusters (Figures 4A and S8).

Integration of the scRNA-seq and the ST datasets

To explore the cell type composition of the tissues analyzed with ST, we leveraged the development of transcriptome deconvolution algorithms. Using a reference dataset,[59] deconvolution tools estimate the proportion of each cell type that contributes to the small bulk cDNA indexed on each ST feature. After comparing multiple software packages (Figure S9), we settled on cell2location, on the basis of a Bayesian model, and explicitly aimed at ST.[60] We supplied cell2location with the reference dataset used for the SingleR analysis described above, allowing us to estimate and compare the proportions of relevant cell types. Both sections originating from the grafted striatum and the host substantia nigra regions were assessed.

After deconvolution of the grafted striatal sections, known cell populations occupy their expected regions in the host tissues (Figure 4F), and the transplant areas clearly stand out from their surroundings. When comparing the distribution of cell types in the transplant area and the surrounding striatal tissues, we find a clear enrichment of dopaminergic neurons, astrocytes, and neuronal progenitor cells (Figures 4C, 4D, and S10A). Part of the transplant population has also been assigned to an inhibitory neuronal population closely related to both septal and ventral midbrain neurons (Figure 4F).

Even though transplants stand out with their human transcript content, not all cells in the transplants are of human origin (Figures 2B and S3). To see how coupled various cell types are to the human origin, we correlated the cell type content with that of human transcripts in features. Dopaminergic neurons showed the highest correlation with human content of the feature, distantly followed by excitatory neurons and neuronal progenitor cells (Figure S10B).

Compared with the scRNA-seq analysis of the transplants, we find large differences in the proportion of cell types (Figures 4E and S10A). The ST deconvolution estimates the number of cells of a specific type in features on the basis of the whole-transcriptome profile matched to a scRNA-seq reference. This deconvolution resulted in the largest fraction of transplanted cells being categorized as dopaminergic neurons with a mean value of 11.3% for all three grafts (95% CI, 10.3%–12.2%; TX[L], 14.5% [95% CI, 13.4%–15.6%]; TX[M], 5.2% [95% CI, 4.2%–6.3%]; TX[S], 5.2% [95% CI, 4.0%–6.3%]). Strikingly, the same cells only accounted for fewer than 1% of cells in the scRNA-seq dataset. VLMCs were enriched in the transplant area in both datasets, but their numbers were much smaller in the ST analysis than in the scRNA-seq. This discrepancy was even more pronounced for the radial glia-like cell population, limited mostly to the subventricular zone in the ST analysis. Neuronal progenitors was the only population that was present in similar proportion in the scRNA-seq dataset and in the ST. However, in the ST dataset, unlike the remaining populations, neuronal progenitor cells are also detectable in low amounts throughout the surrounding tissue.

Comparison of the enriched cell types' proportions within the transplanted striatal to the VM sections also pointed out significant differences (Figures 4B, S11, and S12). Whereas both harbor robust and specific dopaminergic populations, confined to SNpc and ventral tegmental area (VTA) in the VM sections and to the transplant in the striatal sections, VLMCs are enriched to a detectable level only in the choroid plexus epithelium in the midbrain sections and nearly completely missing in the SN and VTA. Thus, presenting a population unique to the transplants. Neuronal progenitor cells were observed in small numbers throughout both section types; however, they were enriched in the grafts, and radial glia-like cells were only sparsely represented in both ventral midbrain and grafted striatal sections.

The largest transplant also allowed us to look at the spatial distribution of different cell types. The dopaminergic neurons tended to be detected closer to the transplant's edges, and VLMCs and astrocytes toward the center of the transplant. This is in line with IHC analyses (Figure 5), which show the same trend in the spatial distribution of the graft. Here, the spatial division of cell types becomes even more evident, with *TH*-positive neurons being positioned at the edges of a graft staining positively for human nuclear antigen and VLMCs identified with *COL1A1* concentrating toward the center.

DISCUSSION

Here, we present a spatial analysis of the composition of hESC-derived dopaminergic transplants grafted in the striatum of a rat model of PD. We also show that despite the number of novel batch correction tools for scRNA-seq,[51–53,61] not all perform equally well on ST. In our dataset, only Seurat has demonstrated reliable performance in most metrics, supporting it as a suitable batch correction algorithm for the exponentially increasing number of ST datasets.

We show that the genes differentially expressed between the transplant and the host striatum or the host tissue in general, featured many dopaminergic markers. These were also observed in abundance in SNpc and did not appear in other morphology-associated clusters, fitting with the expectation. The transplanted DA neurons appear to robustly express all phenotypic markers we would consider to be compatible with a mature dopaminergic signature from a transcriptomic angle. This does not necessarily mean that the neuron has reached full maturity. This requires axonal outgrowth, synaptic

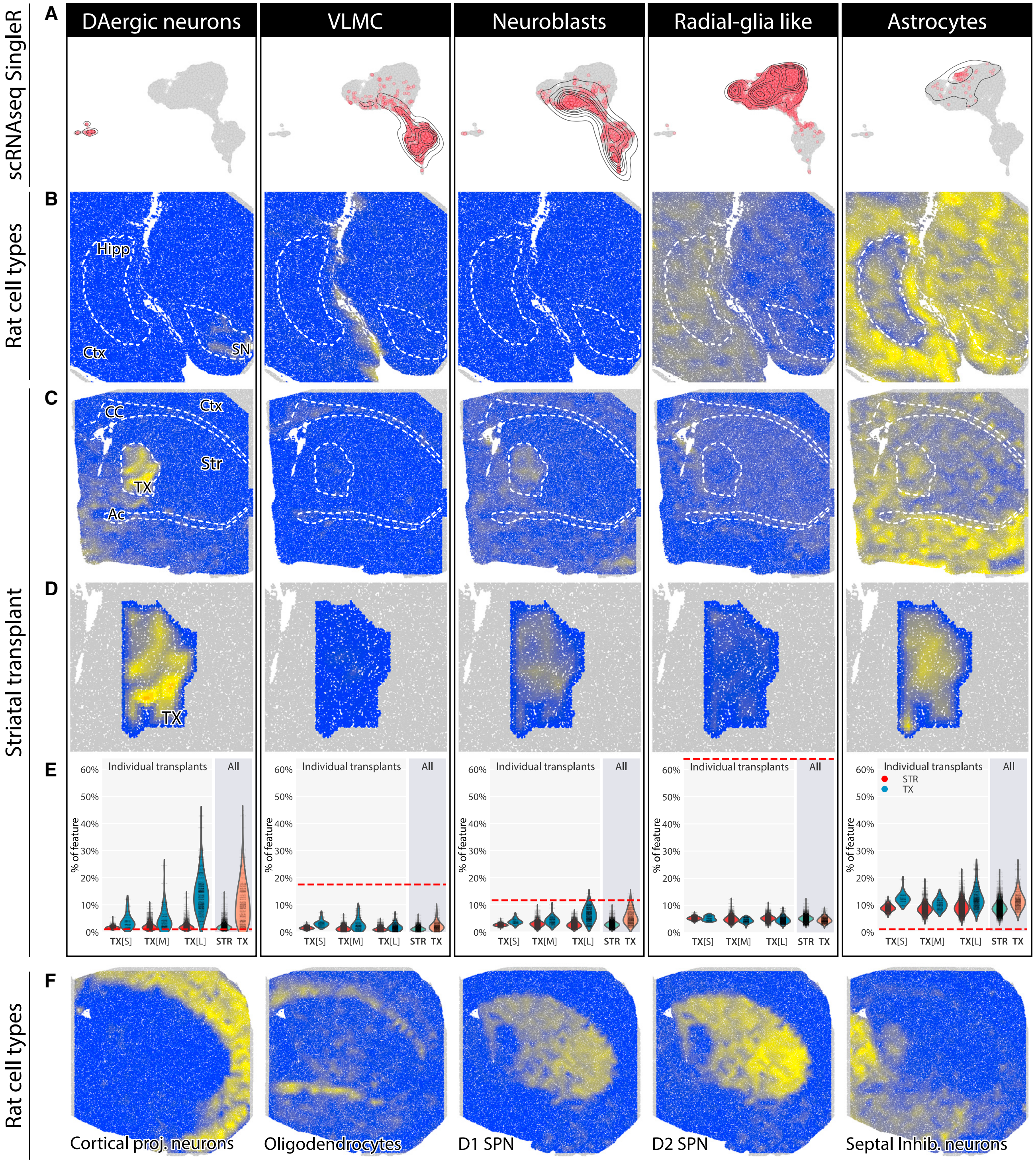

Figure 4. Deconvolution of Spatial Transcriptomics to determine transplant composition *in situ*

(A) Representation of the distribution of the most abundant cell types from the SingleR assignment of the scRNA-seq dataset throughout a UMAP. (B) Deconvolution of a substantia nigra-containing tissue and the distribution of the host cell types. (C) Distribution of cell types in the grafted striatal sections, with a detailed look at the transplant area alone (D). (E) Proportion of cell types in the transplant area versus in the striatum as assessed by deconvolution of the ST slides compared with the proportion of the said cell types in the scRNA-seq dataset (dashed red line signifies their percentage). (F) Distribution of control populations of host cell types throughout the same tissue. Str, striatum; TX, transplant; Ctx, cortex; CC, corpus callosum; Hipp, hippocampus; AC, anterior commissure.

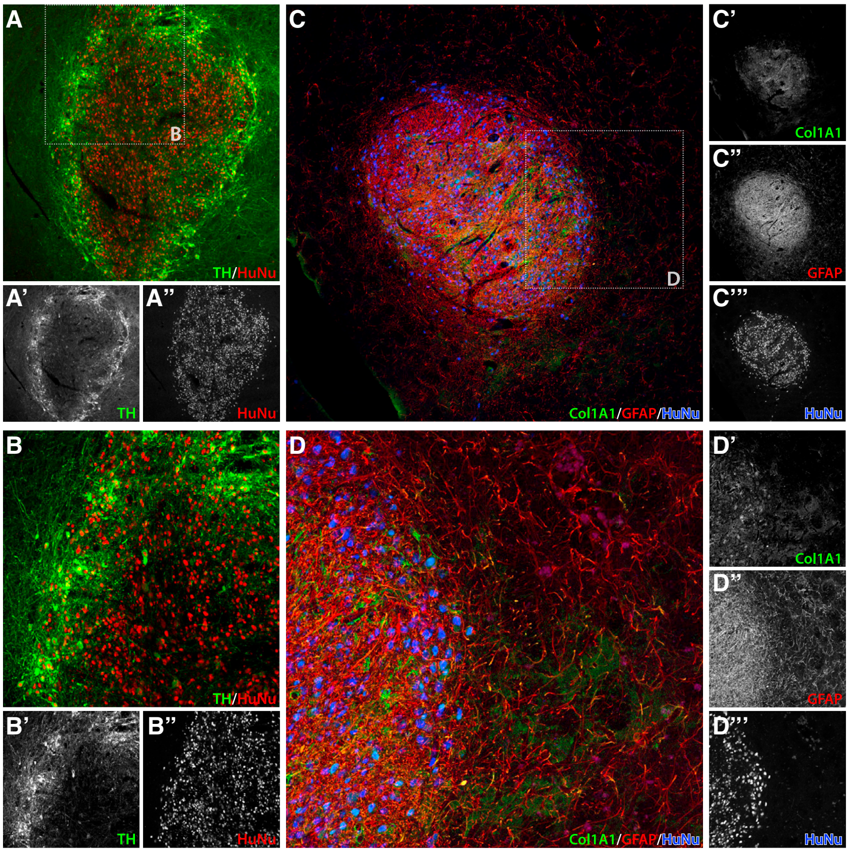

Figure 5. Immunohistological validation of phenotypic markers *in situ* in hESC-derived transplants

IHC of transplants showing (A and B) co-stain of TH (A′ and B′) and HuNu (A″ and B″) for dopaminergic neuron detection and (C and D) co-stain of COL1A1 (C′ and D′), GFAP (C″ and D″) and HuNu (C‴ and D‴) for detection of astrocytes and VLMCs.

interaction, and the adoption of mature membrane potential and firing patterns, all of which may or may not be reflected in the transcriptomic profile. For human DA neurons *in vivo*, the graft can take up to four years to reach full maturity and function.[7] This is impossible to achieve in the rodent brain, but 15 month survival is one of the longest observed to date.

Marker genes for astrocytes and other glial cells were not found to be differentially expressed in the transplant relative to the surrounding rat tissue. However, as these cell types were also represented in host forebrain tissue, their absence is not surprising. This finding supports the uniqueness of the dopaminergic population in the grafted tissue.

Further investigation of the spatial gene distribution reveals the presence of the discovered dopaminergic markers, mainly at the edges of the largest graft. This is in accordance with the IHC analysis, which shows the enrichment of TH-positive cells at the edges of the transplants either due to selective differentiation, migration, or survival.[32,33,62] Relative to dopaminergic markers, VLMC, and astrocyte-associated genes tended to be expressed higher toward the center of the transplants.

Since the advent of deconvolution algorithms, many new software packages have been released. Here we show the difference between stereoscope and cell2location. The results are similar to those previously reported,[61,63] where in most scenarios, cell2location results are more accurate on datasets with known compositions.

We show that ST introduces less bias in cell type composition compared to scRNA-seq.[35,36] This is most likely because there is no tissue dissociation before cDNA synthesis, leading to an equal likelihood of capture for most cell types. This is especially obvious when looking at the vulnerable cell types, such as neurons, which become dwarfed by the glial cell numbers in scRNA-seq. The presented method has a 100 μm resolution, which reports on a larger cell community. Through deconvolution with non-spatial single-cell datasets, the cellular composition of the features and, therefore, the tissue at large can be estimated. The remaining limitations are mainly the uncertainty regarding the cell's position within the feature and the dependency on an accurate and comprehensive single-cell reference dataset. The validity of cell type quantification, generated by deconvolution of the ST data, was confirmed using immunohistochemical analysis, which remains the gold standard for cell quantification but is limited in its multiplexing capacity. With the emergence of higher resolution implementations of this sequencing technique (e.g., with the announced Visium HD or BGI Stereo-seq single-cell resolution platform),[64] many remaining limitations will be removed. However, the analysis pipeline presented here should still be valid and directly applicable. For subcellular resolution, microscopy-based targeted RNA hybridization techniques remain the gold standard.[65] However, they are limited in the number of genes that can be simultaneously detected per cell because of RNA crowding and thus depend on a deep *a priori* knowledge of the assessed tissue.

After ST deconvolution, a large fraction of the transplant is categorized as dopaminergic. The DA cell type also correlated the most with the human transcript content of features. Transplants also harbored astrocyte, VLMC, and neuronal progenitor populations. To facilitate the ST deconvolution, we performed classification at the single-cell level of the scRNA-seq dataset.[36,66] This revealed an additional cell population, the peptidergic inhibitory neurons. These were also observed in low numbers in the transplant areas after ST deconvolution (Figure S10A).

One of the larger observed differences between the scRNA-seq SingleR assignment and the ST analysis was the relative abundance of glial cells. Although they represent the vast majority of the cells recovered with scRNA-seq, they are not as enriched in the transplant area of the ST analysis. The identity of the glial cells also differs between the two analyses. SingleR analysis shows that most of the cells resemble radial glia. However, the gene expression profiles are similar between astrocytes and radial glia in the reference dataset, pointing to the difficulties in dissecting these two populations (Figure S13). The SingleR classification also reveals a rather fluid continuum with multiple cell types overlapping and co-existing in the same UMAP cluster. We believe that this is reflective of the shared origin of the cells and that they retain many transcriptomic similarities. When assessing the *AQP4* gene, specific for astrocytes in the mature but not the developing state,[67,68] we find that even though this is absent in the radial glia of the reference dataset,[59] SingleR still classifies the majority of *AQP4*+ cells as radial glia. This points to a limitation in the SingleR algorithm. Incompatibilities between the differentiated hESCs and the mouse reference could exacerbate the limitations of SingleR. This limitation does not appear to be shared by cell2location,[60] and thus a higher fraction of the features gets classified as astrocytes which also fits with the IHC analysis.

Assessing DA transplants *in situ* also allows the mapping of post-synaptic changes in gene expression. DA depletion in the parkinsonian brain as well as DA replacement therapies, give rise to numerous aberrant changes in gene expression in the GABAergic MSNs.[69] Although *in situ* hybridization has yielded deep insights into individual transcripts and the effect of ectopic DA transplants,[70] ST can provide a holistic assessment of post-synaptic normalization. Interestingly, we here observe a near complete normalization of PENK in the dorsolateral striatum (the principal target of A9 neurons) while still being upregulated in the medial striatum (A10 DA target area) (Figure S6). This does not directly follow the proximity to the graft core, which is here placed in the central and medial striatum. Such gradient has not previously been observed in studies using rat fetal DA transplants.[69,71,72]

In conclusion, this study shows the benefit ST can bring to clinical samples in the future as it deals with several important biases, which can skew our interpretation. It is much easier to dissect pathophysiological changes and brain repair when quantitative transcriptomic data are complemented by spatial information. Furthermore, as the throughput, resolution, and accuracy of these methods have increased exponentially from year to year, there is much that ST methods can elucidate.

MATERIALS AND METHODS

Research animals

All experimental procedures performed in this study were approved by the Ethical Committee to use Laboratory Animals in the Lund-Malmö region. Adult athymic nude female rats were purchased from Envigo (Hsd:RH-Foxn1^{rnu}). The rats were housed in groups of 3 or 4 in individually ventilated cages under a 12 h light/dark cycle with *ad libitum* access to sterile food and water. Seven animals were used in this study. Three animals with histologically verified grafts and three control animals (intact SN) were used for ST. One transplanted animal was retained for IHC. Ten ST arrays were confirmed to contain tissue containing the transplants, five from the largest TX [L], four from the intermediate TX[M], and one from the smallest TX [S], all shown in Figure S2. Seven sections from the intact SN from three animals were used as an internal control for host DA neurons, five of which are shown in Figure S4. For animal details, see Table S1.

6-OHDA lesions

Adult athymic nude female rats were lesioned using a unilateral infusion of 6-OHDA, as previously described.[73] In brief, 3 μL of 6-OHDA (Sigma-Aldrich) at a concentration of 3.5 μg/μL (calculated from freebase weight, HBr-salt in 0.2 mg/ml ascorbic acid in 0.9% sterile saline) was injected using a 30G stainless steel cannula. The cannula was connected via polyethylene tubing to a 10 μL Hamilton syringe at a flow rate of 1 μL/min at the following coordinates (from bregma): anteroposterior (AP), −4.0 mm; mediolateral (ML), −1.3 mm; and dorsoventral (DV) (from dura), −7.0 mm, with the incisor bar set at −4.5 mm. The injection needle was left in place for an additional 3 min to allow diffusion of the toxin.

Drug-induced rotational locomotion

The 6-OHDA lesion was assessed using the drug-induced rotation tests.[74] Rats were placed in automated rotometer bowls modeled after the design of Ungerstedt et al.[75] and recorded using rotation counting software (AccuScan Instruments Inc.). The animals were recorded for 90 min following amphetamine injection (2.5 mg/kg intraperitoneal [i.p.]), and only animals rotating more than 5.5 rpm net clockwise rotations were included in the study.

Cell differentiation and grafting

RC17 human pluripotent stem cells (hPSCs) (Roslin Cells, hPSCreg #RCe021-A) at passage 28 were differentiated and prepared for transplantation, according to.[37,76,77] Cultures of hPSCs were first differentiated into accurate VM progenitor patterning in N2 media with the GSK3 inhibitor CHIR99021 (0.9 μM) and Shh-C24II (300 ng/mL) to obtain the correct VM regionalization in the rostral-caudal axis and floor plate ventralization. At nine days after the start of the differentiation protocol, the cell VM culture was fine-tuned to the caudal early VM progenitors by the addition of FGF8b. At 11 days after the start of differentiation, the cells were replated and expanded in B27 media with FGF8b, AA, and BDNF and cultured for an additional 5 days *in vitro* (DIV) to give rise to late caudal VM progenitors with a high proportion positive for FOXA2/LMX1/OTX2. At 14 DIV, a subset of cells was prepared for mRNA extraction or ICC (validation shown in Aldrin-Kirk et al.[76]). The cultured cells were then prepared for transplantation by dissociation into single-cell suspension in Hank's balanced salt solution (HBSS) plus DNase. The rats were engrafted with 150,000–225,000 differentiated hESCs into the striatum.[65] The cells were engrafted at a concentration of 75,000 cells/μL at the following coordinates (relative to bregma): AP, +0.5 mm; ML, −2.6 mm; and DV (from dura), −4.5 mm, with the incisor bar set at −2.4 mm.

Perfusion and PFA fixation

Animals for IHC analysis were deeply anesthetized by sodium pentobarbital overdose (Apoteksbolaget) and transcardially perfused with a 50 mL physiological saline solution followed by 250 mL of freshly prepared, ice-cold, 4% paraformaldehyde (PFA) in 0.1 M phosphate buffer adjusted to pH 7.4. The brains were then removed and post-fixed for 2 h in cold PFA before storing in 25% buffered sucrose for cryoprotection over at least 24 h until further processing. The remaining PFA fixed brains were cut into 35mm thick coronal sections using a freezing microtome (Leica SM2000R), collected into eight series, and stored in an anti-freeze solution (0.5 M sodium phosphate buffer, 30% glycerol, and 30% ethylene glycol) at -20°C.

IHC post-grafting

Each section used for IHC was washed three times in phosphate buffer saline (PBS) pH 7.4. After the washing step, all sections were submerged for 1 h in a blocking solution consisting of 5% goat serum and 0.25% Triton X-100, in PBS and then incubated with primary antibody overnight at room temperature. The primary antibodies used were: mouse anti-HuNu (1:200; catalog #MAB1281; Merck Millipore), chicken anti-TH (1:1,000; catalog #Ab76442; Abcam), sheep anti-COL1A1 (1:200; catalog #AF6220; R&D Systems), rabbit anti-GFAP (1:1,000; catalog #Z0334; Agilent). Samples were washed three times with PBS to remove unbound primary antibodies and submerged in the blocking solution for 1 h.

The secondary antibody was diluted in the blocking solution and added to sections for 2 h at room temperature. The secondary antibodies used were Alexa Fluor 647 goat anti-mouse (1:400; catalog #A-21236; Invitrogen), Cy3 donkey anti-chicken (1:400; catalog #703-165-155; Jackson ImmunoResearch), Alexa Fluor 488 donkey anti-sheep (1:400; catalog #713-546-147; Jackson ImmunoResearch), and Alexa Fluor 568 goat anti-rabbit (1:400; catalog #A-11011; Invitrogen).

Furthermore, samples were stained with a solution of 1 μg/mL DAPI in PBS to stain for nuclei and washed twice with PBS. After this final step, sections were mounted on coated glass slides, covered with PVA-DABCO, and imaged on a confocal microscope as described below.

Laser scanning confocal microscopy

Confocal imaging was performed using a Leica SP8 microscope. Images were captured using a HyD detector and always with the lasers set to be activated in sequential mode to avoid serial excitation. Solid-state lasers at 405, 448, 552, and 650 nm wavelengths were used to excite their respective fluorophores. A pinhole of 1 AU was always retained during image acquisition. Leica objectives 5×/0.15 and 20×/0.75 were used during imaging acquisition.

Spatial Transcriptomics

All samples were processed according to the published ST protocol,[50] with all the reagents as they are listed in the protocol. In summary, sections were cut with 10 μm thickness and mounted on the active areas of the ST slides. All sections have been fixed with 4% formaldehyde solution and stained with hematoxylin and eosin. Afterward, slides were covered with 85% glycerol, and a cover glass was mounted prior to imaging. The slides were scanned on a slide scanner. Following imaging, sections were permeabilized with pepsin-HCl mixture for 8 min, as determined by the quality control slides ran prior to the main experiment, quickly followed by cDNA synthesis, which took place as described in Salmén et al.[50] overnight at 42°C. This step was followed by second-strand synthesis, after which cDNA was detached from the slide and collected as prescribed.

After the dissociation of cDNA from the slide, probes remaining on the slide were labeled by fluorescent probes and imaged to be able to correct the theoretical feature positions to the actual positions.

The further processing of the detached cDNA was done accordingly with the protocol mentioned above without changes. The indexed library was finally sequenced with NextSeq 500/550 High-Output v2 kit.

Image alignment, detection of spatial spots and tissue

ST Spot Detector was deployed as previously described.[78] H&E tissue image, as well as Cy3-labeled spatial spots image, were supplied, aligned and the precise positions of spatial spots under the tissue were exported.

Sequencing, genome alignment, and data filtering

All samples were sequenced with Illumina NextSeq 500 system using 75 cycle reagents from the NextSeq 500/550 High-Output v2 kit running paired-end protocol: R1 31 bp, i7 6 bp, R2 46 bp. The sequencing data has been processed with STARsolo aligner (version 2.7.9a_2021-06-25).[79] Sequences were aligned to a chimeric (combined) genome where the human (GRCh38 release 93) and rat (Rnor6 release 93) were fused into a double-sized genome. STARsolo was executed with the –solo-MultiMappers EM parameter, which uses the maximum likelihood estimation (MLE)-expectation maximization (EM) algorithm to accurately quantify reads with multi-gene alignment. Feature IDs were provided as a whitelist for cell barcode detection, and deduplication was achieved using the feature unique molecular identifiers (UMIs). The output is a matrix with per feature gene expression read counts. This results in the high fraction of reads being mapped to both human and rat gene homologs as the length of the sequence is frequently insufficient to distinguish between the species. All reads aligning to a single location were allocated to the matching species, remaining reads were proportionally distributed among the genes spanning the matching sites as previously described.[79,80] For species assessment in Figures 2, S3, and S7, genes aligned to more than one location were discarded and only uniquely mapping transcripts were kept. Full details of the STARsolo parameters can be found on the GitHub repository.[79,80]

Ensembl IDs were translated to general symbols to remove the species identity from the transcripts, and duplicate entries and gene isoforms were merged. The resulting gene-feature matrices have been filtered for genes exceeding 2 UMIs in 4 features and features with more than 300 UMIs. The remaining genes and features were discarded.

Feature positions have been detected as described in Salmén et al.,[50] imaged, and processed through ST Spot Detector software[81] without

modifications. The resulting mask of corrected feature positions underneath the tissue was used for the final assessment.

Computational data analysis

Visualization of gene expression

To visualize the presence of genes in the tissue, the tissue images were converted into masks, maximizing the contrast between the tissue and its surroundings. These tissue outlines were then transformed into a set number of features, onto which the gene expression levels were linearly interpolated.

Normalization and batch correction

To assess our baseline, sections from the same morphological areas (hereafter "related sections") were processed without any batch correction. Reads in all features were normalized and log transformed. Three thousand variable genes from all sections were selected for further processing via the Scanpy module. Nearest neighbors were computed (10 neighbors considered) for each feature, and the data were reduced into 2 UMAP dimensions. The division into clusters was computed through Leiden algorithm (resolution = 1).

Seurat was implemented as in Hao et al.[53] with SCT transform.[82] Seurat has integrated related sections on the basis of 3,000 integration features. PCs were kept to the point where further PCs did not explain more than 5% over the previous component, and the dataset was further dimensionally reduced with UMAP. Clusters were assigned shared nearest neighbors (SNNs) through a Leiden algorithm with resolution 1.

Scanorama was implemented in integration with Scanpy.[83] The data were normalized through a Scanpy preprocessing module by scaling the number of reads from each feature to a median number of transcript/features in the dataset and log transformed. Three thousand highly variable genes were selected from (scanpy.pp.highly_variable_genes: flavor = "seurat"), and datasets in this form were corrected with Scanorama (correct_scanpy, default parameters). Nearest neighbors were found for each feature, the output dataset was dimensionally reduced with UMAP, and clusters were assigned by Leiden algorithm with a resolution 1.

DESC was implemented as described in Li et al.,[52] the reads per feature were normalized to 10,000 reads/feature as by the developers' tutorial, log-transformed, and 3,000 highly variable genes were selected. The batch effect was corrected at the level of unique tissues. DESC was trained with default parameters (dimension = [len(genes), 32, 16], tolerance = 0.005, n_neighbours = 3, batch_size = 256, learning_rate = 300, tSNE_core_number = 4) and Louvain resolution of 1.

Transplant selection

Sequencing data were mapped onto human (GRCh38) and rat (Rnor6), with only a single mapping onto the two genomes kept. Counts of all genes belonging to one species within a feature were added up, and the ratio of human to rat genes was carried on into the analysis. Clusters were assigned to features by Seurat, as described previously. The transplant cluster was selected as the cluster with the highest average human transcript proportion in features. Wells with more than 20% of human reads average in clusters made up of host tissues were excluded from joint analyses. The transplant size annotation TX[S], TX[M], and TX [L] was based on the sum of all features belonging to the Seurat clusters with an average of above 15% human reads.

Region annotation

Seurat-assigned clusters were used for region annotation. Clusters were assigned into regions on the basis of the expression of MSN genes for striatum (*PENK*, *ADORA2A*, and *PPP1R1B*), dopaminergic markers for substantia nigra (*TH*, *PBX1*, *SLC6A3*, *ALDH1A1*, *DDC*, and *RET*), *MBP* for white matter tracts and *CCK* and *KCNC2* for cortex. The cluster was categorized into the corresponding region if the average difference of the mean expressions for the genes was larger than 1 standard deviation.

Differential gene expression

For differential gene expression, features from related tissues were assorted into their respective clusters. Mitochondrial and ribosomal genes were removed from the analysis. All features with the same tissue of origin and cluster were merged into a pseudobulk because of reported better performance and lower false discovery rate.[84] All pseudobulk reads were then analyzed with DESeq2.

Deconvolution with a single-cell reference dataset

A reference single-cell dataset for stereoscope and SingleR assessment was selected from a scRNA-seq dataset of the mouse nervous system.[59] The whole dataset was subset for corresponding areas/regions and cell types on the basis of previous knowledge.[77] To prevent low-quality cells from the reference dataset, we removed all cells with fewer than 1,000 transcripts per cell to filter out low-quality cells while preserving less transcriptionally active cell types. Cell labels were assigned based on the level of clusters and description provided by the authors.

The cells were gated at 50–500 cells per cell label for stereoscope deconvolution. In cell categories with more than 500 members, cells to be included in the reference dataset were selected by random choice.

Features were deconvolved with cell2location and stereoscope.[85] With cell2location, substantia nigra and grafted tissues were deconvolved separately, with an estimation of the reference dataset being run for 650 epochs and deconvolution of ST data for 30,000 epochs.

For stereoscope, tissues from each animal were analyzed separately. However, the same reference dataset was used for all. The algorithm was run for 75,000 epochs, as suggested by the authors.

Pearson correlation coefficient was used to correlate cell type content to the human content of a feature.

Previous scRNA-seq dataset of the hESC transplant

Dataset was adapted from Tiklová et al.[36] and analyzed per the authors' instructions. For correlation-based analysis, only clusters

used for cell2location deconvolution were used from the reference dataset.[59]

Cell type assignment of the scRNA-seq reference dataset

To assess the expected prevalence of cell types in a single-cell dataset of hESC-derived dopaminergic transplants from Tiklová et al.[36], the processed scRNA-seq data has been normalized with SCTransform, and the effect of samples has been regressed out. The data and the output have been analyzed with SingleR,[66] using the same reference dataset[59] as cell2location. The reads in this reference dataset have also been normalized with SCTransform with the ChipID variable regressed out.

DATA AVAILABILITY STATEMENT

Sequencing files are available at Sequence Read Archive under the submission number SRA: SUB12409880 with a BioProject identifier SRA: PRJNA914308. The code is available at https://github.com/jana-rajova/ST_hESC_DA_analysis.

SUPPLEMENTAL INFORMATION

Supplemental information can be found online at https://doi.org/10.1016/j.omtm.2023.04.008.

ACKNOWLEDGMENTS

The authors would like to thank Joakim Lundeberg for the valuable discussion and for hosting us for the initial ST experiments. This study was supported by the European Union–funded project NSC-Reconstruct (European Union, H2020, grant agreement [GA] 874758, 2020-23). The research leading to these results has received funding from the New York Stem Cell Foundation, European Research Council (ERC) under ERC grant agreement 771427, Swedish Research Council (2016-00873 and 2020-01684); Knut and Alice Wallenberg Foundation (KAW 2018-0040); Olle Engkvist foundation, The Mats Paulson foundation; Vinnova (2020–04702); Swedish Parkinson Foundation; Swedish Brain Foundation; Strategic Research Area at Lund University Multipark and the Segerfalk Foundation. SSMF, VR (2016-01789), Crafoordska stiftelsen and Åhlensstiftelsen awarded to A.H. M.P. is a New York Stem Cell Foundation Robertson Investigator.

AUTHOR CONTRIBUTIONS

Conceptualization, T.B. and M.P.; Formal Analysis, J.R., T.B., and M.H.; Investigation, J.R., M.A., M.H., S.N., T.C., P.A.-K., and M.D.; Methodology, T.B., M.P., J.R., M.D., and A.H.; Visualization, J.R. and T.B.; Writing – Original Draft, J.R. and T.B.; Writing – Review & Editing, J.R., T.B., M.P., M.Å., M.A., M.D., A.H., P.S., and M.H.

DECLARATION OF INTERESTS

The authors declare no competing interests.

REFERENCES

1. Poewe, W., Seppi, K., Tanner, C.M., Halliday, G.M., Brundin, P., Volkmann, J., Schrag, A.E., and Lang, A.E. (2017). Parkinson disease. Nat. Rev. Dis. Primers *3*, 17013. https://doi.org/10.1038/nrdp.2017.13.
2. LeWitt, P.A., and Fahn, S. (2016). Levodopa therapy for Parkinson disease: a look backward and forward. Neurology *86*, S3–S12. https://doi.org/10.1212/WNL.0000000000002509.
3. PD Med Collaborative Group, Gray, R., Ives, N., Rick, C., Patel, S., Gray, A., Jenkinson, C., McIntosh, E., Wheatley, K., Williams, A., and Clarke, C.E. (2014). Long-term effectiveness of dopamine agonists and monoamine oxidase B inhibitors compared with levodopa as initial treatment for Parkinson's disease (PD MED): a large, open-label, pragmatic randomised trial. Lancet *384*, 1196–1205. https://doi.org/10.1016/S0140-6736(14)60683-8.
4. Freed, C.R., Breeze, R.E., Rosenberg, N.L., Schneck, S.A., Kriek, E., Qi, J.X., Lone, T., Zhang, Y.B., Snyder, J.A., Wells, T.H., et al. (1992). Survival of implanted fetal dopamine cells and neurologic improvement 12 to 46 months after transplantation for Parkinson's disease. N. Engl. J. Med. *327*, 1549–1555. https://doi.org/10.1056/NEJM199211263272202.
5. Widner, H., Tetrud, J., Rehncrona, S., Snow, B., Brundin, P., Gustavii, B., Björklund, A., Lindvall, O., and Langston, J.W. (1992). Bilateral fetal mesencephalic grafting in two patients with parkinsonism induced by 1-methyl-4-phenyl-1,2,3,6-tetrahydropyridine (MPTP). N. Engl. J. Med. *327*, 1556–1563. https://doi.org/10.1056/NEJM199211263272203.
6. Björklund, A., and Parmar, M. (2021). Dopamine cell therapy: from cell replacement to circuitry repair. J. Parkinsons Dis. *11*, S159–S165. https://doi.org/10.3233/JPD-212609.
7. Kefalopoulou, Z., Politis, M., Piccini, P., Mencacci, N., Bhatia, K., Jahanshahi, M., Widner, H., Rehncrona, S., Brundin, P., Björklund, A., et al. (2014). Long-term clinical outcome of fetal cell transplantation for Parkinson disease: two case reports. JAMA Neurol. *71*, 83–87. https://doi.org/10.1001/jamaneurol.2013.4749.
8. Björklund, A., Dunnett, S.B., Brundin, P., Stoessl, A.J., Freed, C.R., Breeze, R.E., Levivier, M., Peschanski, M., Studer, L., and Barker, R. (2003). Neural transplantation for the treatment of Parkinson's disease. Lancet Neurol. *2*, 437–445. https://doi.org/10.1016/s1474-4422(03)00442-3.
9. Barker, R.A., Drouin-Ouellet, J., and Parmar, M. (2015). Cell-based therapies for Parkinson disease-past insights and future potential. Nat. Rev. Neurol. *11*, 492–503. https://doi.org/10.1038/nrneurol.2015.123.
10. Backlund, E.O., Granberg, P.O., Hamberger, B., Knutsson, E., Mårtensson, A., Sedvall, G., Seiger, A., and Olson, L. (1985). Transplantation of adrenal medullary tissue to striatum in parkinsonism. First clinical trials. J. Neurosurg. *62*, 169–173. https://doi.org/10.3171/jns.1985.62.2.0169.
11. Goetz, C.G., Olanow, C.W., Koller, W.C., Penn, R.D., Cahill, D., Morantz, R., Stebbins, G., Tanner, C.M., Klawans, H.L., and Shannon, K.M. (1989). Multicenter study of autologous adrenal medullary transplantation to the corpus striatum in patients with advanced Parkinson's disease. N. Engl. J. Med. *320*, 337–341. https://doi.org/10.1056/NEJM198902093200601.
12. Lindvall, O., Brundin, P., Widner, H., Rehncrona, S., Gustavii, B., Frackowiak, R., Leenders, K.L., Sawle, G., Rothwell, J.C., Marsden, C.D., et al. (1990). Grafts of fetal dopamine neurons survive and improve motor function in Parkinson's disease. Science *247*, 574–577. https://doi.org/10.1126/science.2105529.
13. Clarkson, E.D. (2001). Fetal tissue transplantation for patients with Parkinson's disease: a database of published clinical results. Drugs Aging *18*, 773–785. https://doi.org/10.2165/00002512-200118100-00006.
14. Politis, M., Wu, K., Loane, C., Quinn, N.P., Brooks, D.J., Rehncrona, S., Bjorklund, A., Lindvall, O., and Piccini, P. (2010). Serotonergic neurons mediate dyskinesia side effects in Parkinson's patients with neural transplants. Sci. Transl. Med. *2*, 38ra46. https://doi.org/10.1126/scitranslmed.3000976.
15. Kim, T.W., Koo, S.Y., and Studer, L. (2020). Pluripotent stem cell therapies for Parkinson disease: present challenges and future opportunities. Front. Cell Dev. Biol. *8*, 729. https://doi.org/10.3389/fcell.2020.00729.
16. Steinbeck, J.A., and Studer, L. (2015). Moving stem cells to the clinic: potential and limitations for brain repair. Neuron *86*, 187–206. https://doi.org/10.1016/j.neuron.2015.03.002.
17. Elabi, O.F., Pass, R., Sormonta, I., Nolbrant, S., Drummond, N., Kirkeby, A., Kunath, T., Parmar, M., and Lane, E.L. (2022). Human embryonic stem cell-derived dopaminergic grafts alleviate L-DOPA induced dyskinesia. J. Parkinsons Dis. *12*, 1881–1896. https://doi.org/10.3233/JPD-212920.

18. Kirkeby, A., Nolbrant, S., Tiklova, K., Heuer, A., Kee, N., Cardoso, T., Ottosson, D.R., Lelos, M.J., Rifes, P., Dunnett, S.B., et al. (2017). Predictive markers guide differentiation to improve graft outcome in clinical translation of hESC-based therapy for Parkinson's disease. Cell Stem Cell *20*, 135–148. https://doi.org/10.1016/j.stem.2016.09.004.

19. Xiong, M., Tao, Y., Gao, Q., Feng, B., Yan, W., Zhou, Y., Kotsonis, T.A., Yuan, T., You, Z., Wu, Z., et al. (2021). Human stem cell-derived neurons repair circuits and restore neural function. Cell Stem Cell *28*, 112–126.e6. https://doi.org/10.1016/j.stem.2020.08.014.

20. Brederlau, A., Correia, A.S., Anisimov, S.V., Elmi, M., Paul, G., Roybon, L., Morizane, A., Bergquist, F., Riebe, I., Nannmark, U., et al. (2006). Transplantation of human embryonic stem cell-derived cells to a rat model of Parkinson's disease: effect of in vitro differentiation on graft survival and teratoma formation. Stem Cell. *24*, 1433–1440. https://doi.org/10.1634/stemcells.2005-0393.

21. Schweitzer, J.S., Song, B., Herrington, T.M., Park, T.Y., Lee, N., Ko, S., Jeon, J., Cha, Y., Kim, K., Li, Q., et al. (2020). Personalized iPSC-derived dopamine progenitor cells for Parkinson's disease. N. Engl. J. Med. *382*, 1926–1932. https://doi.org/10.1056/NEJMoa1915872.

22. Morizane, A., Kikuchi, T., Hayashi, T., Mizuma, H., Takara, S., Doi, H., Mawatari, A., Glasser, M.F., Shiina, T., Ishigaki, H., et al. (2017). MHC matching improves engraftment of iPSC-derived neurons in non-human primates. Nat. Commun. *8*, 385. https://doi.org/10.1038/s41467-017-00926-5.

23. Tao, Y., Vermilyea, S.C., Zammit, M., Lu, J., Olsen, M., Metzger, J.M., Yao, L., Chen, Y., Phillips, S., Holden, J.E., et al. (2021). Autologous transplant therapy alleviates motor and depressive behaviors in parkinsonian monkeys. Nat. Med. *27*, 632–639. https://doi.org/10.1038/s41591-021-01257-1.

24. Hallett, P.J., Deleidi, M., Astradsson, A., Smith, G.A., Cooper, O., Osborn, T.M., Sundberg, M., Moore, M.A., Perez-Torres, E., Brownell, A.L., et al. (2015). Successful function of autologous iPSC-derived dopamine neurons following transplantation in a non-human primate model of Parkinson's disease. Cell Stem Cell *16*, 269–274. https://doi.org/10.1016/j.stem.2015.01.018.

25. Adler, A.F., Cardoso, T., Nolbrant, S., Mattsson, B., Hoban, D.B., Jarl, U., Wahlestedt, J.N., Grealish, S., Björklund, A., and Parmar, M. (2019). hESC-derived dopaminergic transplants integrate into basal ganglia circuitry in a preclinical model of Parkinson's disease. Cell Rep. *28*, 3462–3473.e5. https://doi.org/10.1016/j.celrep.2019.08.058.

26. Studer, L., Tabar, V., and McKay, R.D. (1998). Transplantation of expanded mesencephalic precursors leads to recovery in parkinsonian rats. Nat. Neurosci. *1*, 290–295. https://doi.org/10.1038/1105.

27. Kriks, S., Shim, J.W., Piao, J., Ganat, Y.M., Wakeman, D.R., Xie, Z., Carrillo-Reid, L., Auyeung, G., Antonacci, C., Buch, A., et al. (2011). Dopamine neurons derived from human ES cells efficiently engraft in animal models of Parkinson's disease. Nature *480*, 547–551. https://doi.org/10.1038/nature10648.

28. Takagi, Y., Takahashi, J., Saiki, H., Morizane, A., Hayashi, T., Kishi, Y., Fukuda, H., Okamoto, Y., Koyanagi, M., Ideguchi, M., et al. (2005). Dopaminergic neurons generated from monkey embryonic stem cells function in a Parkinson primate model. J. Clin. Invest. *115*, 102–109. https://doi.org/10.1172/JCI21137.

29. Barker, R.A.; TRANSEURO consortium (2019). Designing stem-cell-based dopamine cell replacement trials for Parkinson's disease. Nat. Med. *25*, 1045–1053. https://doi.org/10.1038/s41591-019-0507-2.

30. Takahashi, J. (2020). iPS cell-based therapy for Parkinson's disease: a Kyoto trial. Regen. Ther. *13*, 18–22. https://doi.org/10.1016/j.reth.2020.06.002.

31. Li, W., Englund, E., Widner, H., Mattsson, B., van Westen, D., Lätt, J., Rehncrona, S., Brundin, P., Björklund, A., Lindvall, O., and Li, J.Y. (2016). Extensive graft-derived dopaminergic innervation is maintained 24 years after transplantation in the degenerating parkinsonian brain. Proc. Natl. Acad. Sci. USA *113*, 6544–6549. https://doi.org/10.1073/pnas.1605245113.

32. Bye, C.R., Thompson, L.H., and Parish, C.L. (2012). Birth dating of midbrain dopamine neurons identifies A9 enriched tissue for transplantation into parkinsonian mice. Exp. Neurol. *236*, 58–68. https://doi.org/10.1016/j.expneurol.2012.04.002.

33. Torres, E.M., Dowd, E., and Dunnett, S.B. (2008). Recovery of functional deficits following early donor age ventral mesencephalic grafts in a rat model of Parkinson's disease. Neuroscience *154*, 631–640. https://doi.org/10.1016/j.neuroscience.2008.03.048.

34. de Luzy, I.R., Pavan, C., Moriarty, N., Hunt, C.P.J., Vandenhoven, Z., Khanna, A., Niclis, J.C., Gantner, C.W., Thompson, L.H., and Parish, C.L. (2022). Identifying the optimal developmental age of human pluripotent stem cell-derived midbrain dopaminergic progenitors for transplantation in a rodent model of Parkinson's disease. Exp. Neurol. *358*, 114219. https://doi.org/10.1016/j.expneurol.2022.114219.

35. Xu, P., He, H., Gao, Q., Zhou, Y., Wu, Z., Zhang, X., Sun, L., Hu, G., Guan, Q., You, Z., et al. (2022). Human midbrain dopaminergic neuronal differentiation markers predict cell therapy outcomes in a Parkinson's disease model. J. Clin. Invest. *132*, e156768. https://doi.org/10.1172/JCI156768.

36. Tiklová, K., Nolbrant, S., Fiorenzano, A., Björklund, Å.K., Sharma, Y., Heuer, A., Gillberg, L., Hoban, D.B., Cardoso, T., Adler, A.F., et al. (2020). Single cell transcriptomics identifies stem cell-derived graft composition in a model of Parkinson's disease. Nat. Commun. *11*, 2434. https://doi.org/10.1038/s41467-020-16225-5.

37. Nolbrant, S., Heuer, A., Parmar, M., and Kirkeby, A. (2017). Generation of high-purity human ventral midbrain dopaminergic progenitors for in vitro maturation and intracerebral transplantation. Nat. Protoc. *12*, 1962–1979. https://doi.org/10.1038/nprot.2017.078.

38. Wu, H., Kirita, Y., Donnelly, E.L., and Humphreys, B.D. (2019). Advantages of single-nucleus over single-cell RNA sequencing of adult kidney: rare cell types and novel cell states revealed in fibrosis. J. Am. Soc. Nephrol. *30*, 23–32. https://doi.org/10.1681/ASN.2018090912.

39. Ståhl, P.L., Salmén, F., Vickovic, S., Lundmark, A., Navarro, J.F., Magnusson, J., Giacomello, S., Asp, M., Westholm, J.O., Huss, M., et al. (2016). Visualization and analysis of gene expression in tissue sections by spatial transcriptomics. Science *353*, 78–82. https://doi.org/10.1126/science.aaf2403.

40. Rodriques, S.G., Stickels, R.R., Goeva, A., Martin, C.A., Murray, E., Vanderburg, C.R., Welch, J., Chen, L.M., Chen, F., and Macosko, E.Z. (2019). Slide-seq: a scalable technology for measuring genome-wide expression at high spatial resolution. Science *363*, 1463–1467. https://doi.org/10.1126/science.aaw1219.

41. Marshall, J.L., Noel, T., Wang, Q.S., Chen, H., Murray, E., Subramanian, A., Vernon, K.A., Bazua-Valenti, S., Liguori, K., Keller, K., et al. (2022). High-resolution Slide-seqV2 spatial transcriptomics enables discovery of disease-specific cell neighborhoods and pathways. iScience *25*, 104097. https://doi.org/10.1016/j.isci.2022.104097.

42. Ke, R., Mignardi, M., Pacureanu, A., Svedlund, J., Botling, J., Wählby, C., and Nilsson, M. (2013). In situ sequencing for RNA analysis in preserved tissue and cells. Nat. Methods *10*, 857–860. https://doi.org/10.1038/nmeth.2563.

43. Chen, X., Sun, Y.C., Church, G.M., Lee, J.H., and Zador, A.M. (2018). Efficient in situ barcode sequencing using padlock probe-based BaristaSeq. Nucleic Acids Res. *46*, e22. https://doi.org/10.1093/nar/gkx1206.

44. Wang, X., Allen, W.E., Wright, M.A., Sylwestrak, E.L., Samusik, N., Vesuna, S., Evans, K., Liu, C., Ramakrishnan, C., Liu, J., et al. (2018). Three-dimensional intact-tissue sequencing of single-cell transcriptional states. Science *361*, eaat5691. https://doi.org/10.1126/science.aat5691.

45. Liu, S., Punthambaker, S., Iyer, E.P.R., Ferrante, T., Goodwin, D., Fürth, D., Pawlowski, A.C., Jindal, K., Tam, J.M., Mifflin, L., et al. (2021). Barcoded oligonucleotides ligated on RNA amplified for multiplexed and parallel in situ analyses. Nucleic Acids Res. *49*, e58. https://doi.org/10.1093/nar/gkab120.

46. Thrane, K., Eriksson, H., Maaskola, J., Hansson, J., and Lundeberg, J. (2018). Spatially resolved transcriptomics enables dissection of genetic heterogeneity in stage III cutaneous malignant melanoma. Cancer Res. *78*, 5970–5979. https://doi.org/10.1158/0008-5472.CAN-18-0747.

47. Kuppe, C., Ramirez Flores, R.O., Li, Z., Hayat, S., Levinson, R.T., Liao, X., Hannani, M.T., Tanevski, J., Wünnemann, F., Nagai, J.S., et al. (2022). Spatial multi-omic map of human myocardial infarction. Nature *608*, 766–777. https://doi.org/10.1038/s41586-022-05060-x.

48. Erickson, A., He, M., Berglund, E., Marklund, M., Mirzazadeh, R., Schultz, N., Kvastad, L., Andersson, A., Bergenstråhle, L., Bergenstråhle, J., et al. (2022). Spatially resolved clonal copy number alterations in benign and malignant tissue. Nature *608*, 360–367. https://doi.org/10.1038/s41586-022-05023-2.

49. Ray, P.R., Shiers, S., Caruso, J.P., Tavares-Ferreira, D., Sankaranarayanan, I., Uhelski, M.L., Li, Y., North, R.Y., Tatsui, C., Dussor, G., et al. (2023). RNA profiling of human dorsal root ganglia reveals sex-differences in mechanisms promoting neuropathic pain. Brain *146*, 749–766. https://doi.org/10.1093/brain/awac266.

50. Salmén, F., Ståhl, P.L., Mollbrink, A., Navarro, J.F., Vickovic, S., Frisén, J., and Lundeberg, J. (2018). Barcoded solid-phase RNA capture for Spatial Transcriptomics

profiling in mammalian tissue sections. Nat. Protoc. *13*, 2501–2534. https://doi.org/10.1038/s41596-018-0045-2.

51. Hie, B., Bryson, B., and Berger, B. (2019). Efficient integration of heterogeneous single-cell transcriptomes using Scanorama. Nat. Biotechnol. *37*, 685–691. https://doi.org/10.1038/s41587-019-0113-3.

52. Li, X., Wang, K., Lyu, Y., Pan, H., Zhang, J., Stambolian, D., Susztak, K., Reilly, M.P., Hu, G., and Li, M. (2020). Deep learning enables accurate clustering with batch effect removal in single-cell RNA-seq analysis. Nat. Commun. *11*, 2338. https://doi.org/10.1038/s41467-020-15851-3.

53. Hao, Y., Hao, S., Andersen-Nissen, E., Mauck, W.M., 3rd, Zheng, S., Butler, A., Lee, M.J., Wilk, A.J., Darby, C., Zager, M., et al. (2021). Integrated analysis of multimodal single-cell data. Cell *184*, 3573–3587.e29. https://doi.org/10.1016/j.cell.2021.04.048.

54. Bergenstråhle, J., Larsson, L., and Lundeberg, J. (2020). Seamless integration of image and molecular analysis for spatial transcriptomics workflows. BMC Genom. *21*, 482. https://doi.org/10.1186/s12864-020-06832-3.

55. Paxinos (2017). The Rat Brain in Stereotaxic Coordinates (Academic Press).

56. Gokce, O., Stanley, G.M., Treutlein, B., Neff, N.F., Camp, J.G., Malenka, R.C., Rothwell, P.E., Fuccillo, M.V., Südhof, T.C., and Quake, S.R. (2016). Cellular taxonomy of the mouse striatum as revealed by single-cell RNA-seq. Cell Rep. *16*, 1126–1137. https://doi.org/10.1016/j.celrep.2016.06.059.

57. Carta, M., Carlsson, T., Muñoz, A., Kirik, D., and Björklund, A. (2010). Role of serotonin neurons in the induction of levodopa- and graft-induced dyskinesias in Parkinson's disease. Mov. Disord. *25* (*Suppl 1*), S174–S179. https://doi.org/10.1002/mds.22792.

58. Aldrin-Kirk, P., Heuer, A., Wang, G., Mattsson, B., Lundblad, M., Parmar, M., and Björklund, T. (2016). DREADD modulation of transplanted DA neurons reveals a novel parkinsonian dyskinesia mechanism mediated by the serotonin 5-HT6 receptor. Neuron *90*, 955–968. https://doi.org/10.1016/j.neuron.2016.04.017.

59. Zeisel, A., Hochgerner, H., Lönnerberg, P., Johnsson, A., Memic, F., van der Zwan, J., Häring, M., Braun, E., Borm, L.E., La Manno, G., et al. (2018). Molecular architecture of the mouse nervous system. Cell *174*, 999–1014.e22. https://doi.org/10.1016/j.cell.2018.06.021.

60. Kleshchevnikov, V., Shmatko, A., Dann, E., Aivazidis, A., King, H.W., Li, T., Elmentaite, R., Lomakin, A., Kedlian, V., Gayoso, A., et al. (2022). Cell2location maps fine-grained cell types in spatial transcriptomics. Nat. Biotechnol. *40*, 661–671. https://doi.org/10.1038/s41587-021-01139-4.

61. Li, B., Zhang, W., Guo, C., Xu, H., Li, L., Fang, M., Hu, Y., Zhang, X., Yao, X., Tang, M., et al. (2022). Benchmarking spatial and single-cell transcriptomics integration methods for transcript distribution prediction and cell type deconvolution. Nat. Methods *19*, 662–670. https://doi.org/10.1038/s41592-022-01480-9.

62. Jönsson, M.E., Ono, Y., Björklund, A., and Thompson, L.H. (2009). Identification of transplantable dopamine neuron precursors at different stages of midbrain neurogenesis. Exp. Neurol. *219*, 341–354. https://doi.org/10.1016/j.expneurol.2009.06.006.

63. Ma, Y., and Zhou, X. (2022). Spatially informed cell-type deconvolution for spatial transcriptomics. Nat. Biotechnol. *40*, 1349–1359. https://doi.org/10.1038/s41587-022-01273-7.

64. Chen, A., Liao, S., Cheng, M., Ma, K., Wu, L., Lai, Y., Qiu, X., Yang, J., Xu, J., Hao, S., et al. (2022). Spatiotemporal transcriptomic atlas of mouse organogenesis using DNA nanoball-patterned arrays. Cell *185*, 1777–1792.e21. https://doi.org/10.1016/j.cell.2022.04.003.

65. Grealish, S., Heuer, A., Cardoso, T., Kirkeby, A., Jönsson, M., Johansson, J., Björklund, A., Jakobsson, J., and Parmar, M. (2015). Monosynaptic tracing using modified rabies virus reveals early and extensive circuit integration of human embryonic stem cell-derived neurons. Stem Cell Rep. *4*, 975–983. https://doi.org/10.1016/j.stemcr.2015.04.011.

66. Aran, D., Looney, A.P., Liu, L., Wu, E., Fong, V., Hsu, A., Chak, S., Naikawadi, R.P., Wolters, P.J., Abate, A.R., et al. (2019). Reference-based analysis of lung single-cell sequencing reveals a transitional profibrotic macrophage. Nat. Immunol. *20*, 163–172. https://doi.org/10.1038/s41590-018-0276-y.

67. Fallier-Becker, P., Vollmer, J.P., Bauer, H.C., Noell, S., Wolburg, H., and Mack, A.F. (2014). Onset of aquaporin-4 expression in the developing mouse brain. Int. J. Dev. Neurosci. *36*, 81–89. https://doi.org/10.1016/j.ijdevneu.2014.06.001.

68. Castañeyra-Ruiz, L., González-Marrero, I., Hernández-Abad, L.G., Carmona-Calero, E.M., Pardo, M.R., Baz-Davila, R., Lee, S., Muhonen, M., Borges, R., and Castañeyra-Perdomo, A. (2022). AQP4 labels a subpopulation of white matter-dependent glial radial cells affected by pediatric hydrocephalus, and its expression increased in glial microvesicles released to the cerebrospinal fluid in obstructive hydrocephalus. Acta Neuropathol. Commun. *10*, 41. https://doi.org/10.1186/s40478-022-01345-4.

69. Cenci, M.A., Campbell, K., and Björklund, A. (1993). Neuropeptide messenger RNA expression in the 6-hydroxydopamine-lesioned rat striatum reinnervated by fetal dopaminergic transplants: differential effects of the grafts on preproenkephalin, preprotachykinin and prodynorphin messenger RNA levels. Neuroscience *57*, 275–296. https://doi.org/10.1016/0306-4522(93)90062-k.

70. Björklund, A. (1992). Dopaminergic transplants in experimental parkinsonism: cellular mechanisms of graft-induced functional recovery. Curr. Opin. Neurobiol. *2*, 683–689. https://doi.org/10.1016/0959-4388(92)90039-n.

71. Cadet, J.L., Zhu, S.M., and Angulo, J.A. (1991). Intrastriatal implants of fetal mesencephalic cells attenuate the increases in striatal proenkephalin mRNA observed after unilateral 6-hydroxydopamine-induced lesions of the striatum. Brain Res. Bull. *27*, 707–711. https://doi.org/10.1016/0361-9230(91)90050-t.

72. Mendez, I.M., Naus, C.C., Elisevich, K., and Flumerfelt, B.A. (1993). Normalization of striatal proenkephalin and preprotachykinin mRNA expression by fetal substantia nigra grafts. Exp. Neurol. *119*, 1–10. https://doi.org/10.1006/exnr.1993.1001.

73. Heuer, A., Lelos, M.J., Kelly, C.M., Torres, E.M., and Dunnett, S.B. (2013). Dopamine-rich grafts alleviate deficits in contralateral response space induced by extensive dopamine depletion in rats. Exp. Neurol. *247*, 485–495. https://doi.org/10.1016/j.expneurol.2013.01.020.

74. Ungerstedt, U. (1976). 6-hydroxydopamine-induced degeneration of the nigrostriatal dopamine pathway: the turning syndrome. Pharmacol. Ther. B. *2*, 37–40. https://doi.org/10.1016/0306-039x(76)90016-7.

75. Ungerstedt, U., and Arbuthnott, G.W. (1970). Quantitative recording of rotational behavior in rats after 6-hydroxy-dopamine lesions of the nigrostriatal dopamine system. Brain Res. *24*, 485–493. https://doi.org/10.1016/0006-8993(70)90187-3.

76. Aldrin-Kirk, P., Åkerblom, M., Cardoso, T., Nolbrant, S., Adler, A.F., Liu, X., Heuer, A., Davidsson, M., Parmar, M., and Björklund, T. (2022). A novel two-factor monosynaptic TRIO tracing method for assessment of circuit integration of hESC-derived dopamine transplants. Stem Cell Rep. *17*, 159–172. https://doi.org/10.1016/j.stemcr.2021.11.014.

77. Kirkeby, A., Grealish, S., Wolf, D.A., Nelander, J., Wood, J., Lundblad, M., Lindvall, O., and Parmar, M. (2012). Generation of regionally specified neural progenitors and functional neurons from human embryonic stem cells under defined conditions. Cell Rep. *1*, 703–714. https://doi.org/10.1016/j.celrep.2012.04.009.

78. (2018). ST Spot Detector. https://github.com/SpatialTranscriptomicsResearch/st_spot_detector.

79. Kaminow, B., Yunusov, D., and Dobin, A. (2021). STARsolo: accurate, fast and versatile mapping/quantification of single-cell and single-nucleus RNA-seq data. Preprint at bioRxiv. https://doi.org/10.1101/2021.05.05.442755.

80. STARsolo (2022). https://github.com/alexdobin/STAR.

81. Wong, K., Navarro, J.F., Bergenstråhle, L., Ståhl, P.L., and Lundeberg, J. (2018). ST Spot Detector: a web-based application for automatic spot and tissue detection for spatial Transcriptomics image datasets. Bioinformatics *34*, 1966–1968. https://doi.org/10.1093/bioinformatics/bty030.

82. Hafemeister, C., and Satija, R. (2019). Normalization and variance stabilization of single-cell RNA-seq data using regularized negative binomial regression. Genome Biol. *20*, 296. https://doi.org/10.1186/s13059-019-1874-1.

83. Wolf, F.A., Angerer, P., and Theis, F.J. (2018). SCANPY: large-scale single-cell gene expression data analysis. Genome Biol. *19*, 15. https://doi.org/10.1186/s13059-017-1382-0.

84. Squair, J.W., Gautier, M., Kathe, C., Anderson, M.A., James, N.D., Hutson, T.H., Hudelle, R., Qaiser, T., Matson, K.J.E., Barraud, Q., et al. (2021). Confronting false discoveries in single-cell differential expression. Nat. Commun. *12*, 5692. https://doi.org/10.1038/s41467-021-25960-2.

85. Andersson, A., Bergenstråhle, J., Asp, M., Bergenstråhle, L., Jurek, A., Fernández Navarro, J., and Lundeberg, J. (2020). Single-cell and spatial transcriptomics enables probabilistic inference of cell type topography. Commun. Biol. *3*, 565. https://doi.org/10.1038/s42003-020-01247-y.

OMTM, Volume *29*

Supplemental information

Deconvolution of spatial sequencing provides accurate characterization of hESC-derived DA transplants *in vivo*

Jana Rájová, Marcus Davidsson, Martino Avallone, Morgan Hartnor, Patrick Aldrin-Kirk, Tiago Cardoso, Sara Nolbrant, Annelie Mollbrink, Petter Storm, Andreas Heuer, Malin Parmar, and Tomas Björklund

Table S1

Supplemental Table 1: Description of used tissue sections and their animal/tissue of origin

Sample name	Animal signifier	Organism	Breed	Trans-plant age (months)	Sex	Tissue	Cell line	Cell line type
TX[L.1]	TX[L]	Rattus norvegicus + Homo sapiens	athymic nude rat	6	Female	Brain (striatal section with a transplant)	RC17	hESC derived
TX[L.2]	TX[L]	Rattus norvegicus + Homo sapiens	athymic nude rat	6	Female	Brain (striatal section with a transplant)	RC17	hESC derived
TX[L.3]	TX[L]	Rattus norvegicus + Homo sapiens	athymic nude rat	6	Female	Brain (striatal section with a transplant)	RC17	hESC derived
TX[L.4]	TX[L]	Rattus norvegicus + Homo sapiens	athymic nude rat	6	Female	Brain (striatal section with a transplant)	RC17	hESC derived
TX[L.5]	TX[L]	Rattus norvegicus + Homo sapiens	athymic nude rat	6	Female	Brain (striatal section with a transplant)	RC17	hESC derived
TX[M.1]	TX[M]	Rattus norvegicus + Homo sapiens	athymic nude rat	13	Female	Brain (striatal section with a transplant)	RC17	hESC derived
TX[M.2]	TX[M]	Rattus norvegicus + Homo sapiens	athymic nude rat	13	Female	Brain (striatal section with a transplant)	RC17	hESC derived
TX[M.3]	TX[M]	Rattus norvegicus + Homo sapiens	athymic nude rat	13	Female	Brain (striatal section with a transplant)	RC17	hESC derived
TX[M.4]	TX[M]	Rattus norvegicus + Homo sapiens	athymic nude rat	13	Female	Brain (striatal section with a transplant)	RC17	hESC derived
TX[S]	TX[S]	Rattus norvegicus + Homo sapiens	athymic nude rat	15	Female	Brain (striatal section with a transplant)	RC17	hESC derived
SN[1.1]	SN[1]	Rattus norvegicus	athymic nude rat	NA	Female	Brain (midbrain section with substantia nigra)	NA	NA
SN[1.2]	SN[1]	Rattus norvegicus	athymic nude rat	NA	Female	Brain (midbrain section with substantia nigra)	NA	NA
SN[2.1]	SN[2]	Rattus norvegicus	athymic nude rat	NA	Female	Brain (midbrain section with substantia nigra + cortex)	NA	NA
SN[2.2]	SN[2]	Rattus norvegicus	athymic nude rat	NA	Female	Brain (midbrain section with substantia nigra + cortex)	NA	NA
SN[2.3]	SN[2]	Rattus norvegicus	athymic nude rat	NA	Female	Brain (midbrain section with substantia nigra + cortex)	NA	NA
SN[3.1]	SN[3]	Rattus norvegicus	Sprague-Dawley	NA	Female	Brain (midbrain section with substantia nigra)	NA	NA
SN[3.2]	SN[3]	Rattus norvegicus	Sprague-Dawley	NA	Female	Brain (midbrain section with substantia nigra)	NA	NA
IHC analysis	TX[4]	Rattus norvegicus + Homo sapiens	athymic nude rat	15	Female	Brain (striatal section with a transplant)	RC17	hESC derived

Figure S1

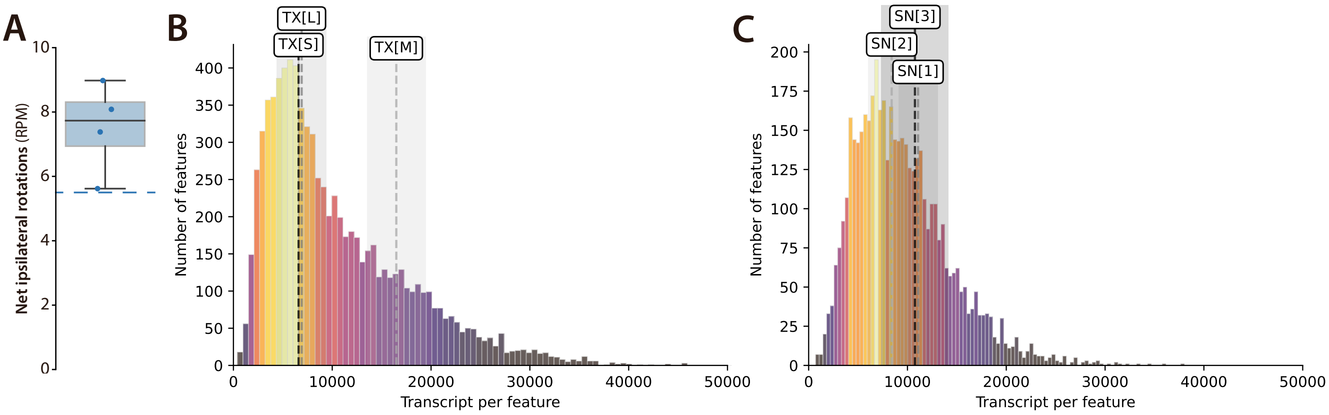

Figure S1: (a) d-Amphetamine-induced rotation of 6-OHDA lesioned animals prior to transplantation. Dashed line represents the inclusion cut-off. (b-c) Histograms of transcript count distribution per feature in (b) grafted tissue sections (TX[**S**], TX[**M**], TX[**L**]) and (c) the substantia nigra sections. Dashed lines denote sample mean per animal and the gray box one standard deviation from that mean.

Figure S2

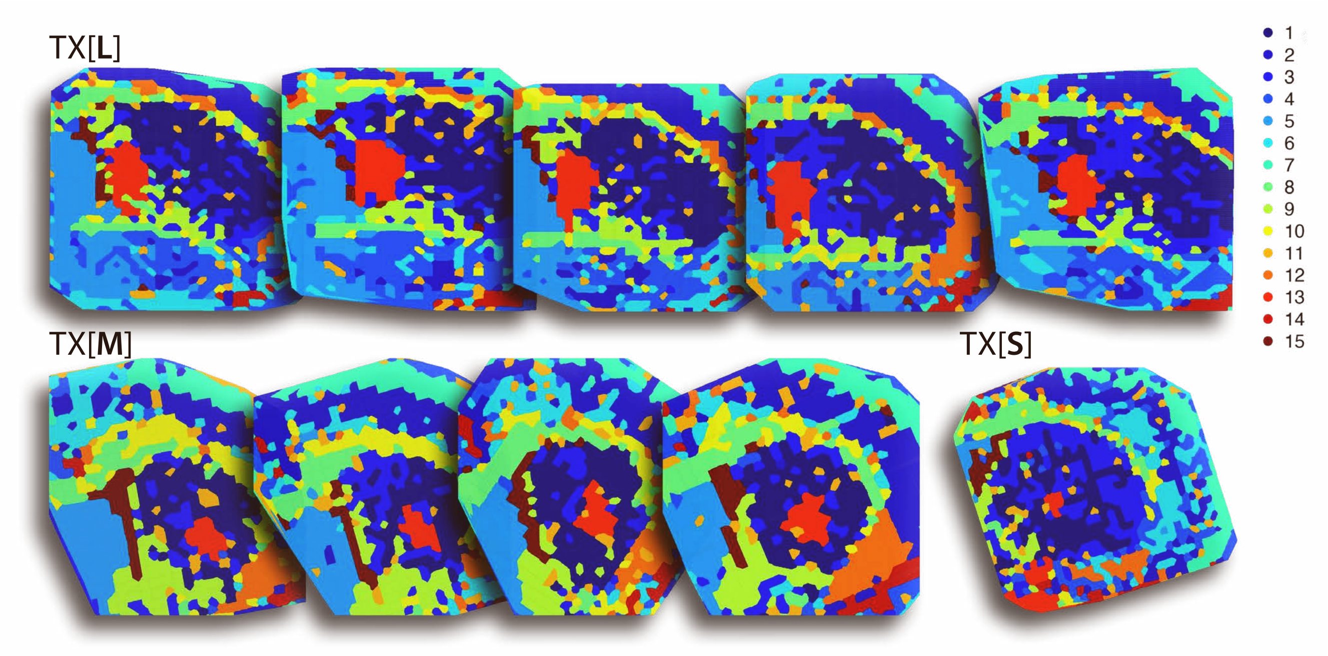

Figure S2: Seurat cluster assignment for grafted tissue sections (TX[**S**], TX[**M**], TX[**L**])

Figure S3

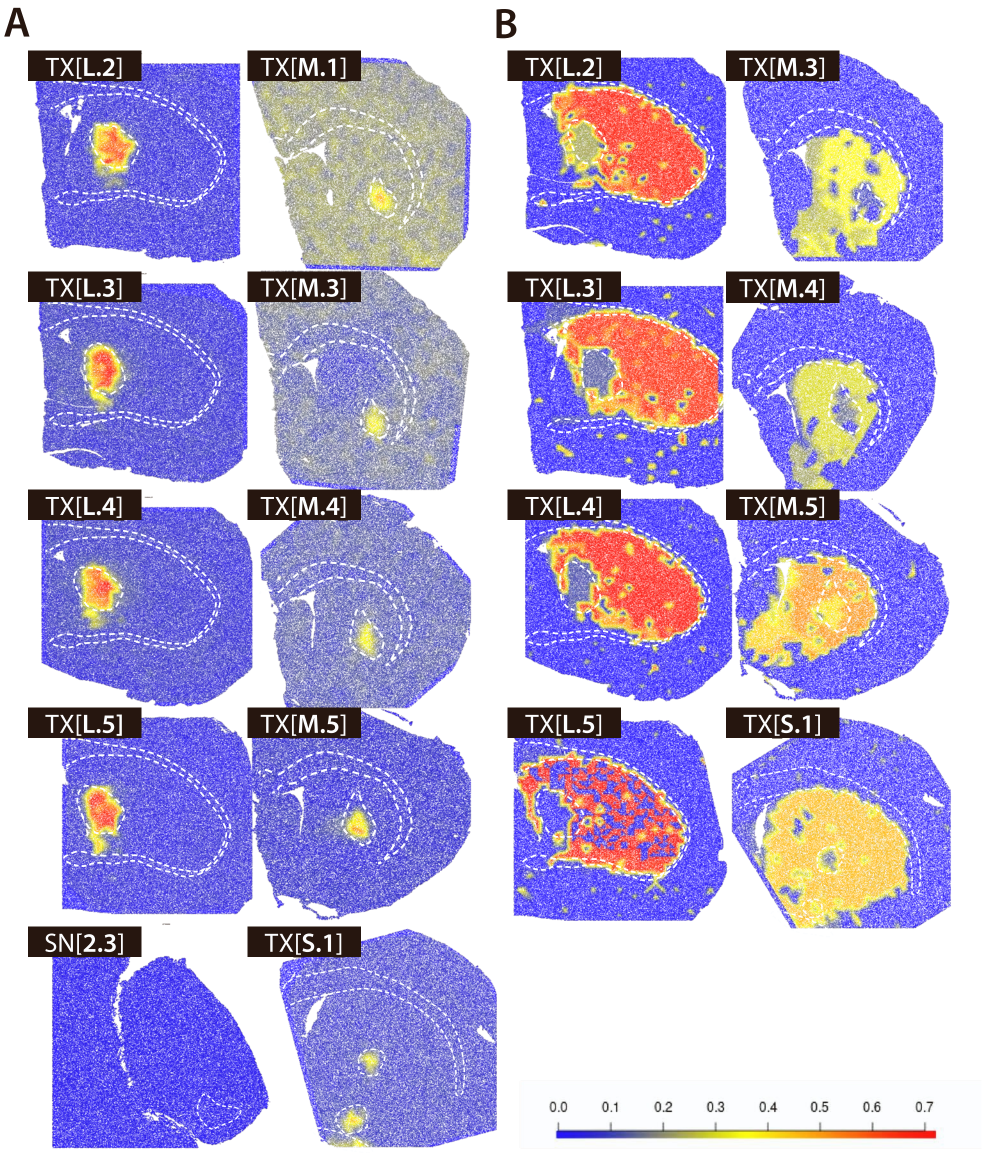

Figure S3: (a) Proportion of transcripts aligning to a unique site on the human genome in grafted striatal tissues. (b) Striatal score for forebrain tissue sections as assigned by cumulative expression of PENK, ADORA2A and PPP1R1B MSN marker genes.

Figure S4

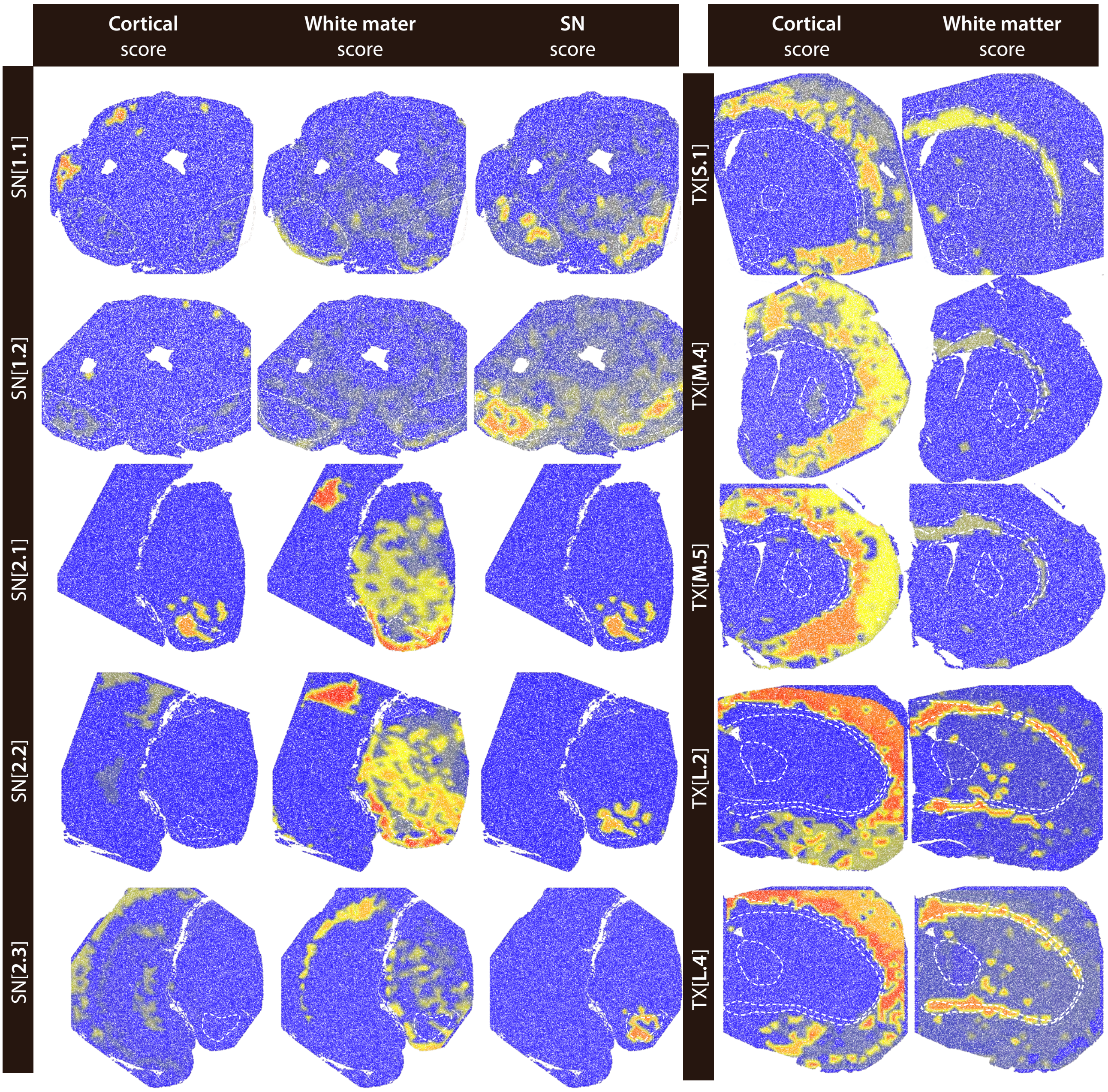

Figure S4: Region assignment through cumulative marker gene expression scores. Shown scores of Seurat-inferred clusters for expression of cortical, white matter or substantia nigra related genes in midbrain (SN[1-3]) and forebrain (TX[S-L]) sections. Dashed white guidelines are provided as guidelines for substantia nigra regions in midbrain sections and white matter tracts and grafted areas in forebrain sections.

Figure S5

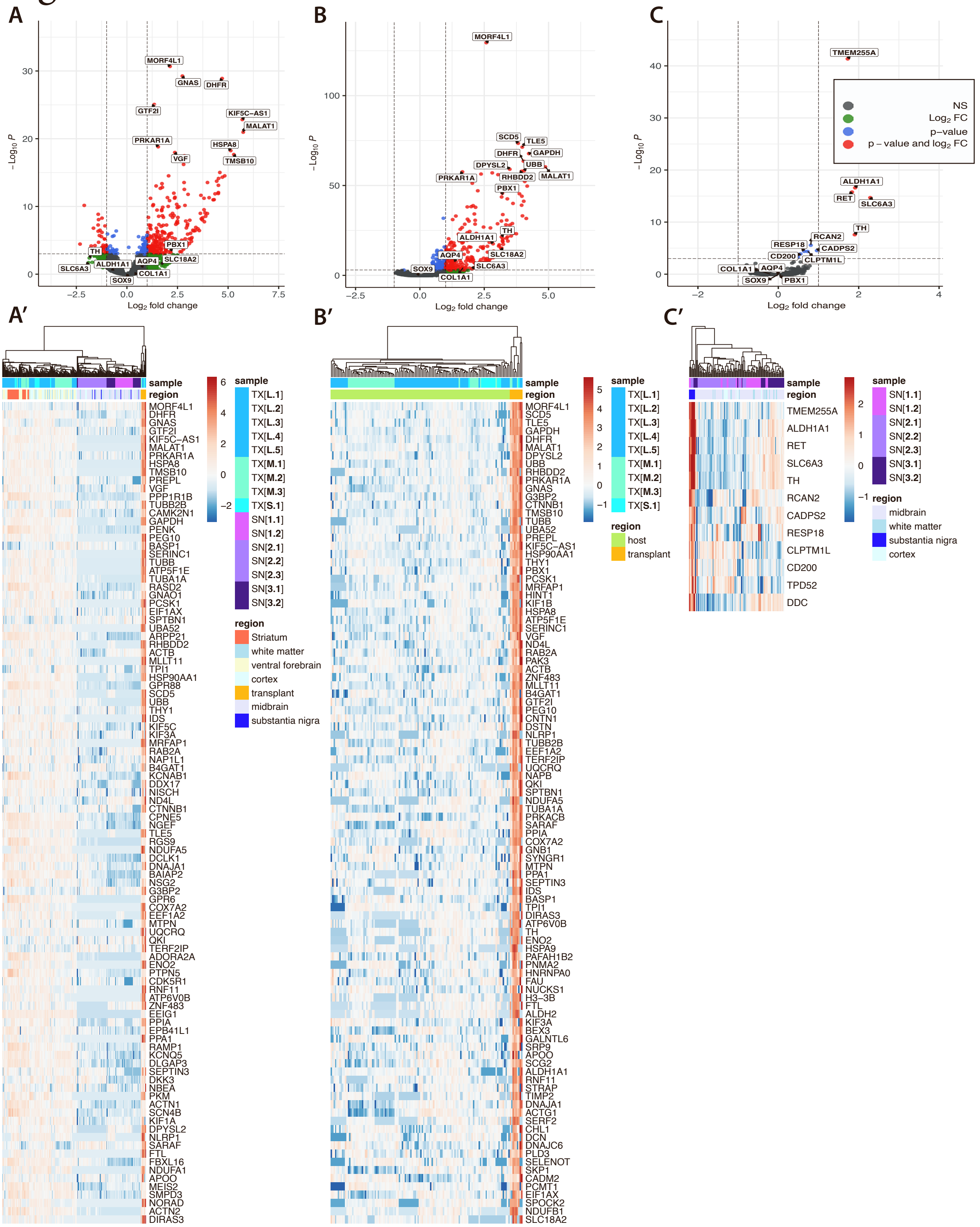

Figure S5: (a) Differentially expressed genes between the transplant and SN regions. Shown 10 genes with highest log2fold score and marker genes for dopaminergic neurons, VLMCs and mature astrocytes. (a') Top 100 genes with the highest log2fold change between the transplant and substantia nigra. Their scaled expression in other tissues is shown for comparison. (b) Differentially expressed genes between host tissues in the grafted forebrain regions and the transplants. (b') Top 100 genes with the highest log2fold change between the transplant and the remaining host tissue in the grafted forebrain sections. (c) Differentially expressed genes between the SN and the surrounding midbrain tissue. (c') Top 100 genes with the highest log2fold change between the SN and the surrounding midbrain tissue.

Figure S6

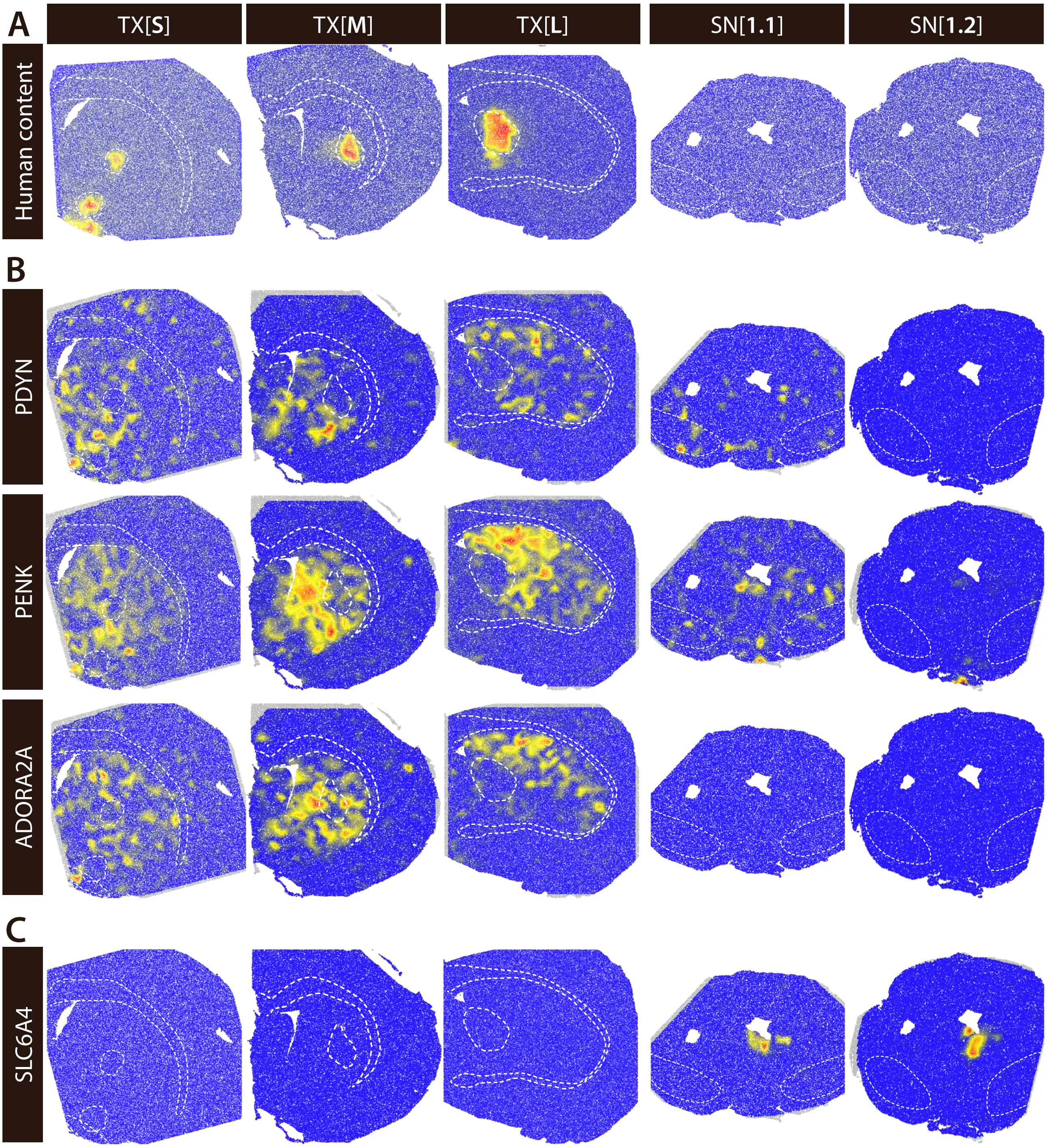

Figure S6: (a) Comparison of human transcript-rich areas in the grafted tissues and the human-assigned transcript content in the control midbrain tissues. (b) Striatal markers PDYN, PENK and ADORA2A expression in striatal tissues compared to the midbrain sections. (c) The expression of serotonin receptor SLC6A4 in the transplanted sections as compared to the midbrain sections, where SLC6A4 is present in the periaquedactal gray area. Dashed white guidelines are provided as guidelines for substantia nigra (SN) regions in midbrain sections and white matter tracts and grafted areas in forebrain sections.

Figure S7

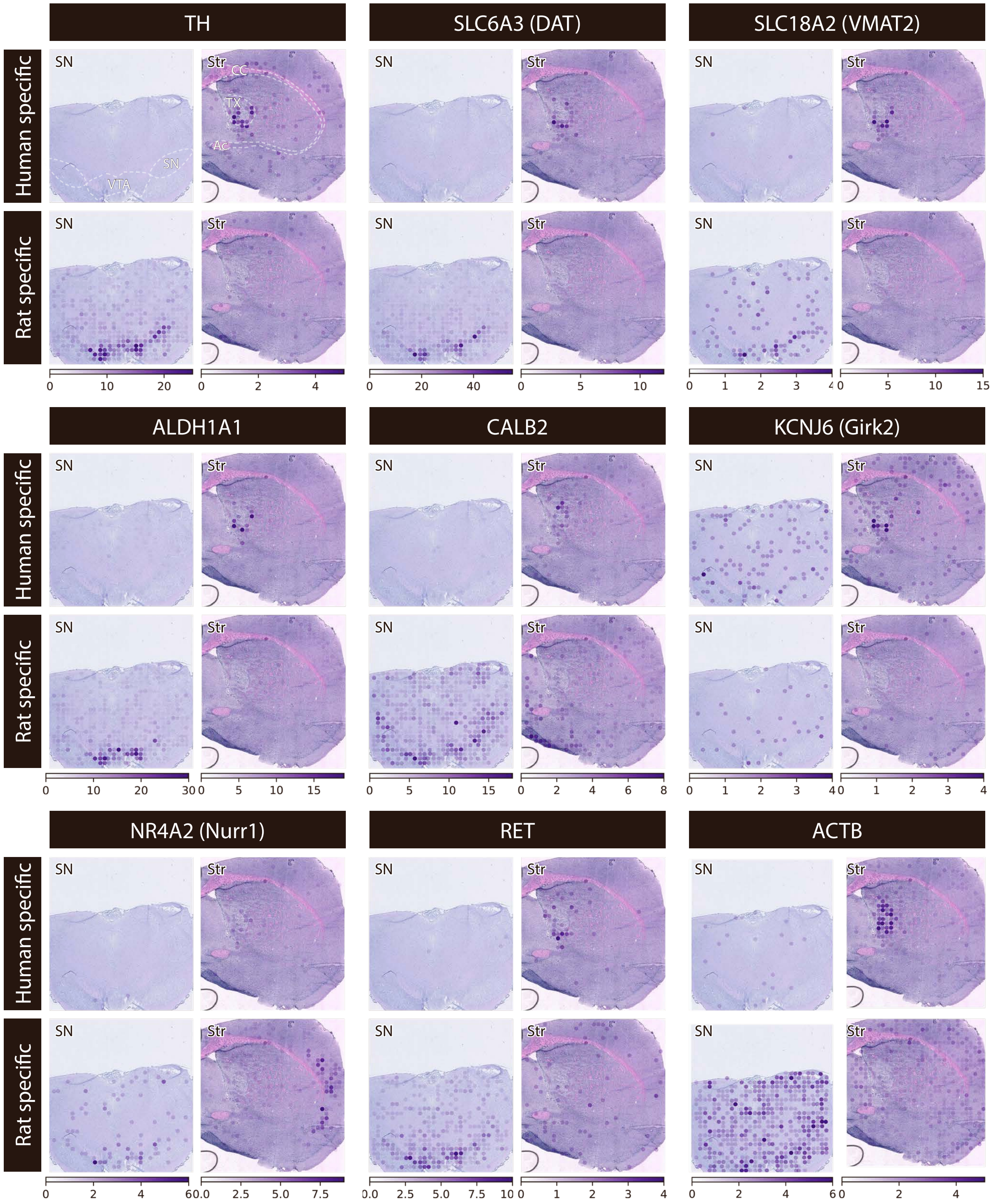

Figure S7: Expression of species specific transcripts for DA neuron marker genes and beta-actin (ACTB) in a rat SN section and grafted striatal section. VTA - ventral tegmental area, SN - substantia nigra, CC - corpus callosum, TX - transplant, Ac - anterior commisure.

Figure S8

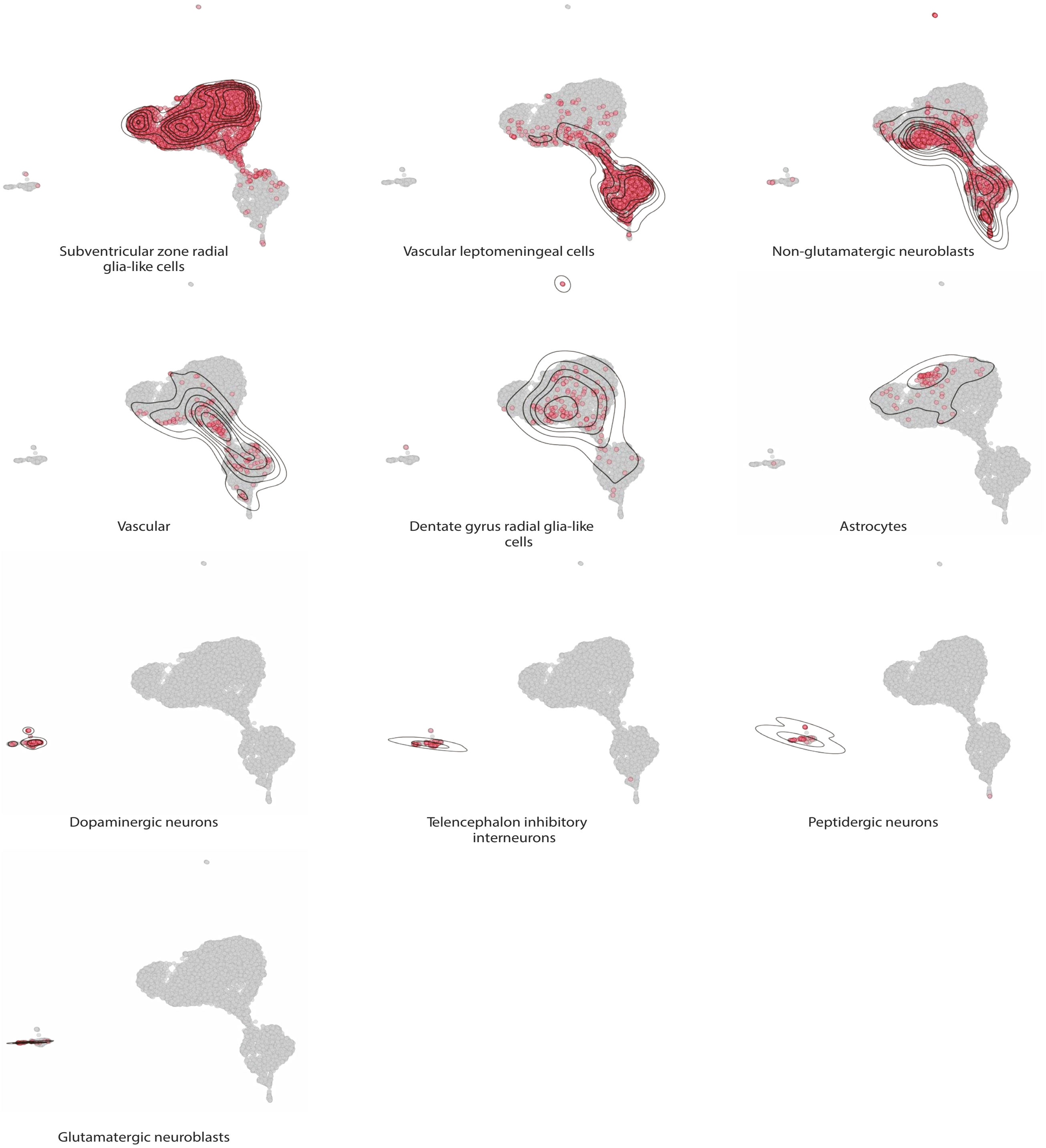

Figure S8: SingleR assignment of cells from a dopaminergic transplant using scRNAseq analysis. Presented are all cell types that were assigned more than 10 cells ordered from the most to least abundant. Contour plots show the density of cells assigned to UMAP space.

Figure S9

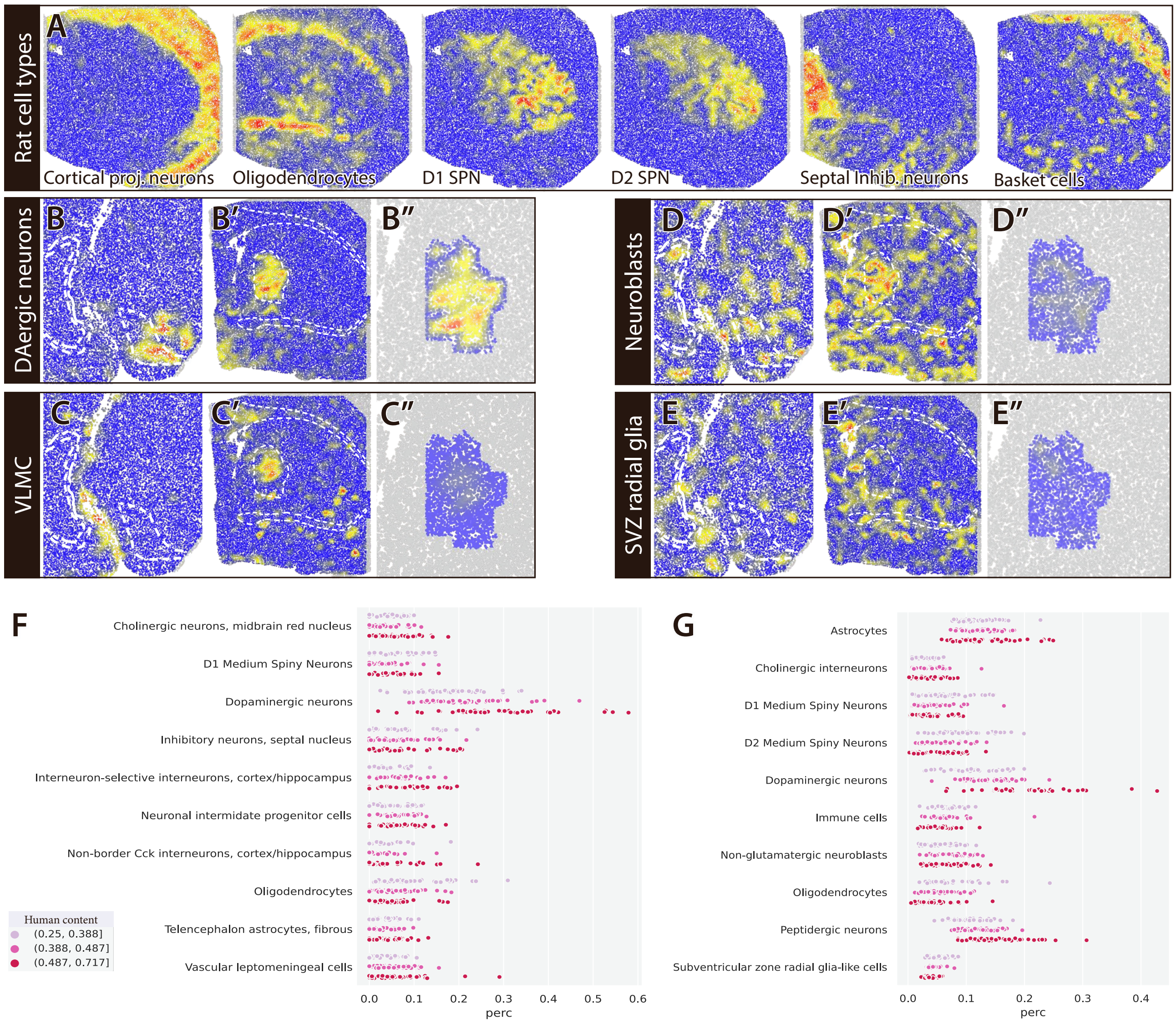

Figure S9: (a-e”) Deconvolution of grafted striatal and midbrain tissue sections with stereoscope. (a) Presence of host cell types. (b-e”) Presence of cell types previously observed in hESC-derived dopaminergic transplants. Shown is deconvolved cell type representation in midbrain sections with highlighted substantia nigra region (b-e), grafted striatal tissues (b’-e’) and only the transplant area (b”-e”). (f) Representation of cell types in features with increasing proportions of human reads as analyzed with stereoscope. (g) Representation of cell types in features with increasing proportions of human reads as analyzed with cell2location.

Figure S10

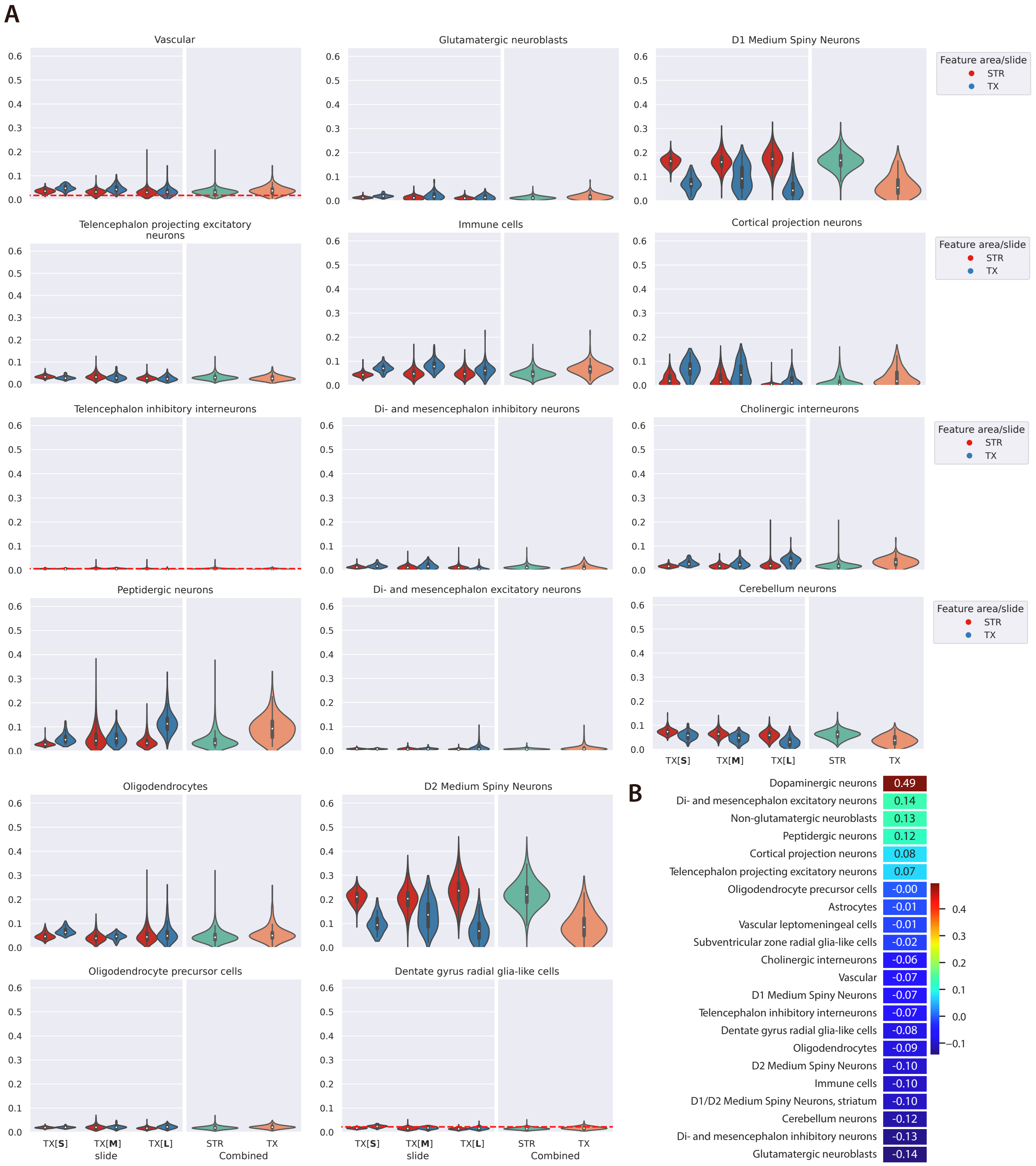

Figure S10: (a) Estimated proportions of reference dataset cell types in striatal and TX assigned features. Red line signifies cell type proportion of a cell type assigned to more than 10 cells scRNAseq analysis of hESC dopaminergic transplants as assigned by SingleR. (b) Correlations of proportions of human transcripts in a feature to a cell type content.

Figure S11

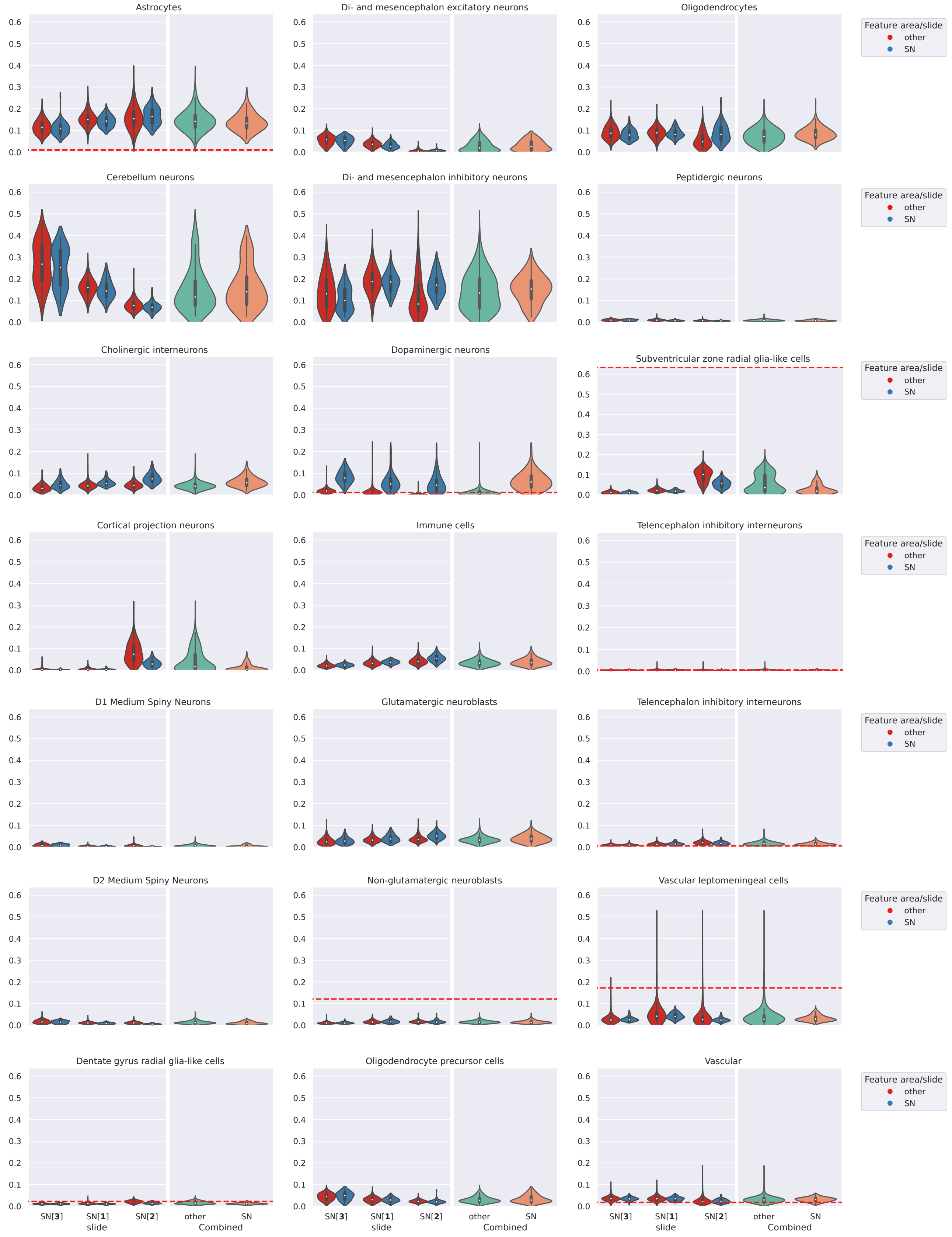

Figure S11: Proportion of cell types in substantia nigra as compared to the remainder of midbrain regions. Red line corresponds to the proportion of a cell type in scRNAseq analysis of dopaminergic transplants as assigned by SingleR

Figure S12

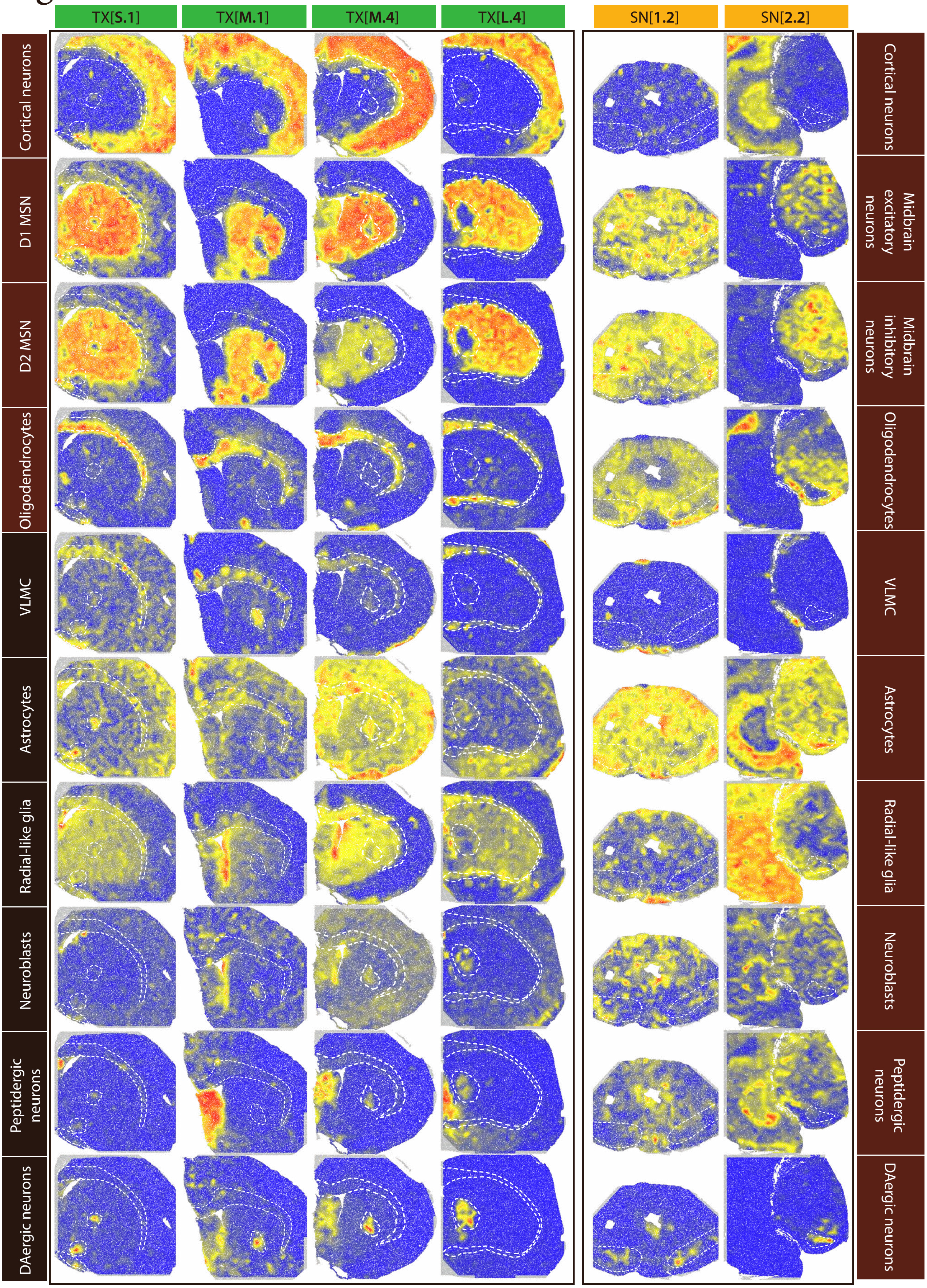

Figure S12: Relative distribution of cell types in representative transplanted sections versus in midbrain sections with substantia nigra region. Host cell types are marked with red and cells found in human transplants with black background. Dashed white guidelines are provided as guidelines for substantia nigra regions in midbrain sections and white matter tracts and grafted areas in forebrain sections.

Figure S13

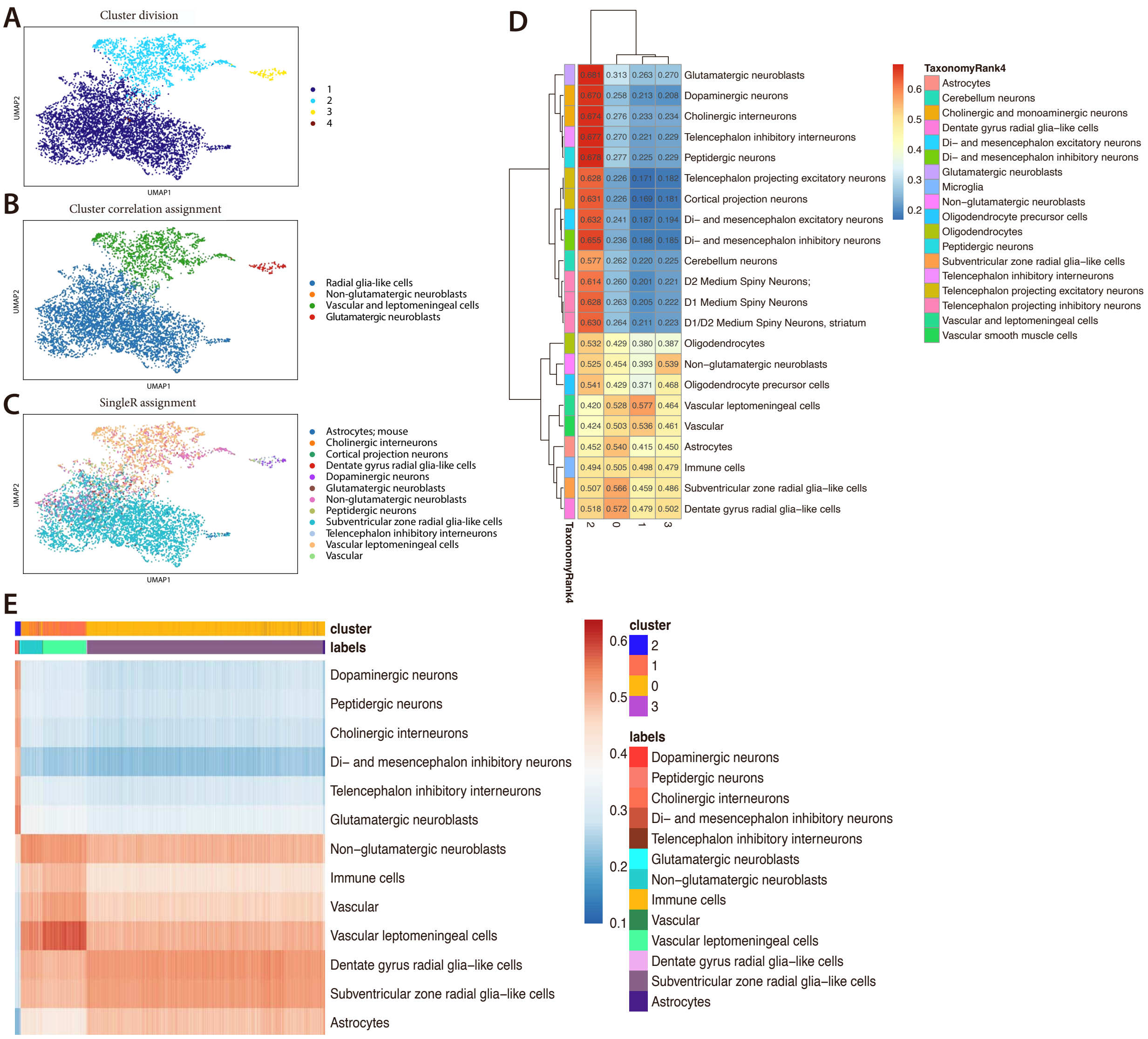

Figure S13: (a-c) UMAP representation of hESC derived dopaminergic graft scRNAseq analysis annotated by (a) cluster assignment, (b) correlation of clusters with the reference dataset cell types and (c) SingleR assigned cell types using the same reference dataset as in (d) and (e). (d) Spearmann correlation of clusters' transcriptome with that of the reference dataset cell types'. (e) All cell type scores for SingleR annotation of hESC derived dopaminergic graft scRNAseq analysis.
